# GhPYL9-5D and GhPYR1-3 A positively regulate Arabidopsis and cotton responses to ABA, drought, high salinity and osmotic stress

**DOI:** 10.1186/s12870-023-04330-8

**Published:** 2023-06-10

**Authors:** Yibin Wang, Gaofeng Zhang, Huimin Zhou, Shanshan Yin, Yunxiang Li, Caixia Ma, Pengyun Chen, Lirong Sun, Fushun Hao

**Affiliations:** National Key Laboratory of Cotton Bio-breeding and Integrated Utilization, School of Life Sciences, College of Agriculture, Henan University, Kaifeng, 475004 China

**Keywords:** Cotton, *Arabidopsis*, ABA receptor protein, ABA response, Abiotic stress, Stomatal closure

## Abstract

**Background:**

Abscisic acid (ABA) receptor pyrabactin resistance 1/PYR1-like/regulatory components of ABA receptor proteins (PYR/PYL/RCARs) have been demonstrated to play pivotal roles in ABA signaling and in response to diverse environmental stimuli including drought, salinity and osmotic stress in *Arabidopsis*. However, whether and how GhPYL9-5D and GhPYR1-3A, the homologues of *Arabidopsis* PYL9 and PYR1 in cotton, function in responding to ABA and abiotic stresses are still unclear.

**Results:**

GhPYL9-5D and GhPYR1-3A were targeted to the cytoplasm and nucleus. Overexpression of *GhPYL9-5D* and *GhPYR1-3A* in *Arabidopsis* wild type and sextuple mutant *pyr1pyl1pyl2pyl4pyl5pyl8* plants resulted in ABA hypersensitivity in terms of seed germination, root growth and stomatal closure, as well as seedling tolerance to water deficit, salt and osmotic stress. Moreover, the VIGS (Virus-induced gene silencing) cotton plants, in which *GhPYL9-5D* or *GhPYR1-3A* were knocked down, showed clearly reduced tolerance to polyethylene glycol 6000 (PEG)-induced drought, salinity and osmotic stresses compared with the controls. Additionally, transcriptomic data revealed that *GhPYL9-5D* was highly expressed in the root, and *GhPYR1-3A* was strongly expressed in the fiber and stem. *GhPYL9-5D*, *GhPYR1-3A* and their homologs in cotton were highly expressed after treatment with PEG or NaCl, and the two genes were co-expressed with redox signaling components, transcription factors and auxin signal components. These results suggest that GhPYL9-5D and GhPYR1-3A may serve important roles through interplaying with hormone and other signaling components in cotton adaptation to salt or osmotic stress.

**Conclusions:**

GhPYL9-5D and GhPYR1-3A positively regulate ABA-mediated seed germination, primary root growth and stomatal closure, as well as tolerance to drought, salt and osmotic stresses likely through affecting the expression of multiple downstream stress-associated genes in *Arabidopsis* and cotton.

**Supplementary Information:**

The online version contains supplementary material available at 10.1186/s12870-023-04330-8.

## Background

Phytohormone abscisic acid (ABA) plays pivotal roles in regulating many growth and development processes including seed dormancy and germination, seedling growth, and leaf senescence in plants. Moreover, it controls stomatal movement and plant responses to diverse stresses such as drought, salinity and extreme temperature [1–5]. When plants are subjected to adverse environmental stimuli, particularly water scarcity, ABA levels in tissues evidently elevate by *de novo* biosynthesis and/or hydrolysis of the glucose-conjugated ABA. The ABA then binds to its receptors, and activates ABA signaling components to induce the expression of numerous stress-responsive genes, helping plants to deal with the stresses. For example, ABA promotes root growth to facilitate water absorption, and triggers stomatal closure to prevent water loss under drought stress [3, 4, 6].

Currently, main ABA signal components and signal transduction mechanisms have been uncovered in *Arabidopsis*. The key ABA signaling components include pyrabactin resistance1/PYR1-like/regulatory components of ABA receptor proteins (AtPYR/PYL/RCARs, hereafter named as AtPYLs for simplicity), clade A type 2 C protein phosphatases (AtPP2Cs), sucrose nonfermenting (SNF) 1-related kinases 2s (AtSnRK2s), and ABA-responsive transcription factors. In the presence of ABA, ABA binds to AtPYLs, enabling the latter to interact with and suppress the activity of AtPP2Cs. Consequently, AtPP2Cs unbind and release AtSnRK2s. The AtSnRK2s are then activated, and phosphorylate downstream transcription factors, further triggering the transcription of a number of functional genes and causing relevant physiological responses [3, 4].

Plant PYLs belong to the steroidogenic acute regulatory protein-related lipid-transfer superfamily with a ligand-binding pocket enclosed by four conserved loops, CL1-CL4 [7, 8]. There exist 14 AtPYL members (AtPYR1 and 13 AtPYLs) in *Arabidopsis* [7, 8]. Of these, AtPYR1, AtPYL1, AtPYL2 and AtPYL3 repress the activities of AtPP2Cs in ABA-dependent patterns while AtPYL4, AtPYL5, AtPYL6, AtPYL8, AtPYL9, AtPYL10 and AtPYL13 show the ABA-independent inhibition of AtPP2C activities [9–11]. It has been demonstrated that nearly all of the AtPYR1 and AtPYLs function redundantly in ABA-modulated seed germination, root development, leaf senescence, flowering, seed production and stomatal closure, and in response to drought and osmotic stress although AtPYL3, AtPYL7, AtPYL11 and AtPYL12 serve minor roles relative to other members in these processes [8, 12–23].

PYLs in other plant species except *Arabidopsis* are also implicated in ABA-mediated growth and development processes, and in responsiveness to multiple stresses including water deficit, salinity and osmotic stress. For instance, mutations in rice *OsPYL1/2/3/4/5/6/12* or *OsPYL7/8/9/10/11/13* caused notably increased seed germination rates in the presence of 2.5, 5 and 10 µM ABA. The high-order mutant *ospyl1/2/3/4/5/6/12* showed less sensitivity to ABA in term of seedling growth than wild type (WT). Moreover, *ospyl1/4/6* mutant plants were more sensitive to drought stress than WT, and *ospyl1/2/3/4/5/6/12* mutant displayed clear insensitivity to ABA-induced stomatal closure compared with WT [24]. It has been addressed that overexpression of *OsPYL3*, *OsPYL5*, *OsPYL6*, *OsPYL9* or *OsPYL11* in rice resulted in markedly enhanced ABA sensitivity during seed germination and/or seedling growth. Similar results were obtained in the transgenic plants overexpressing tomato PYL genes 6g050500 and 3g007310 in *Arabidopsis*, maize *ZmPYL3, ZmPYL9, ZmPYL10* and *ZmPYL13* in *Arabidopsis*, *SlPYL9* in tomato, *TaPYL4* in wheat, grape *VyPYL9* and poplar *PePYL6* and *PePYL9* in *Arabidopsis* [25–33]. Moreover, transgenic rice lines overexpressing *OsPYL3*, *OsPYL5*, *OsPYL/RCAR7*, *OsPYL9*, *OsPYL10* or *OsPYL11* exhibited elevated drought tolerance compared with the control. Similar findings were reported in the transgenic plants overexpressing *AaPYL9* in *A. annua*, tomato genes 6g050500 and 3g007310 in *Arabidopsis*, *ZmPYL8*, *ZmPYL9* and *ZmPYL12* in *Arabidopsis*, *PtPYRL1* and *PtPYRL5* in *Arabidopsis* and poplar, *SlPYL9* in tomato, *TaPYL4* in wheat, *VyPYL9* in *Arabidopsis*, and *MdPYL9* in apple [26–31, 34–38]. Additionally, overexpression of *OsPYL5* caused prominently enhanced salt resistance [36], and *OsPYL6* overexpression leaded to clearly increased mannitol repression of seedling growth in an *indica* rice cv. Pusa Sugandh 2 [32]. In contrast, overexpression of *PtPYRL1* and *PtPYRL5* in poplar improved the osmotic resistance of the transgenic plants [39].

Cotton (*Gossypium hirsutum* L.) is the most natural fiber crop in the world. Its growth and development are frequently threatened by adverse environmental stresses such as drought and high salinity. Therefore, it is imperative for researchers to investigate the strategies to improve the stress tolerance of cotton plants applying the gene transformation technology. PYL genes may be good candidates for the goal. In *G. hirsutum*, 40 GhPYLs were identified. Most of them have been addressed to interact with GhPP2C members in vitro. The expression of many *GhPYLs* markedly enhanced after treatment with ABA, salt or osmotic stresses [40]. Moreover, transgenic lines overexpressing *GhPYL9-11 A* and *GhPYL10/12/26* in *Arabidopsis* were hypersensitive to ABA in terms of seed germination and early seedling growth compared with the controls [41, 42]. Overexpression of *GhPYL10/12/26* also conferred enhanced drought tolerance in *Arabidopsis* [41], and ectopic expression of *GhPYL9-11A* in *Arabidopsis* resulted in increased sensitivity to osmotic stress during seedling growth [42]. However, the functions of other GhPYLs members in cotton are largely unknown to date.

In this study, we investigated the roles of GhPYL9-5D (GH_D12G2568) and GhPYR1-3A (GH_A12G2288), and found that they positively modulated ABA-mediated seed germination, primary root elongation and stomatal closure, as well as tolerance to drought, salt and osmotic stresses in *Arabidopsis* and cotton.

## Results

### Both GhPYL9-5D and GhPYR1-3A were targeted to the cytoplasm and nucleus

**Fig. 1:**
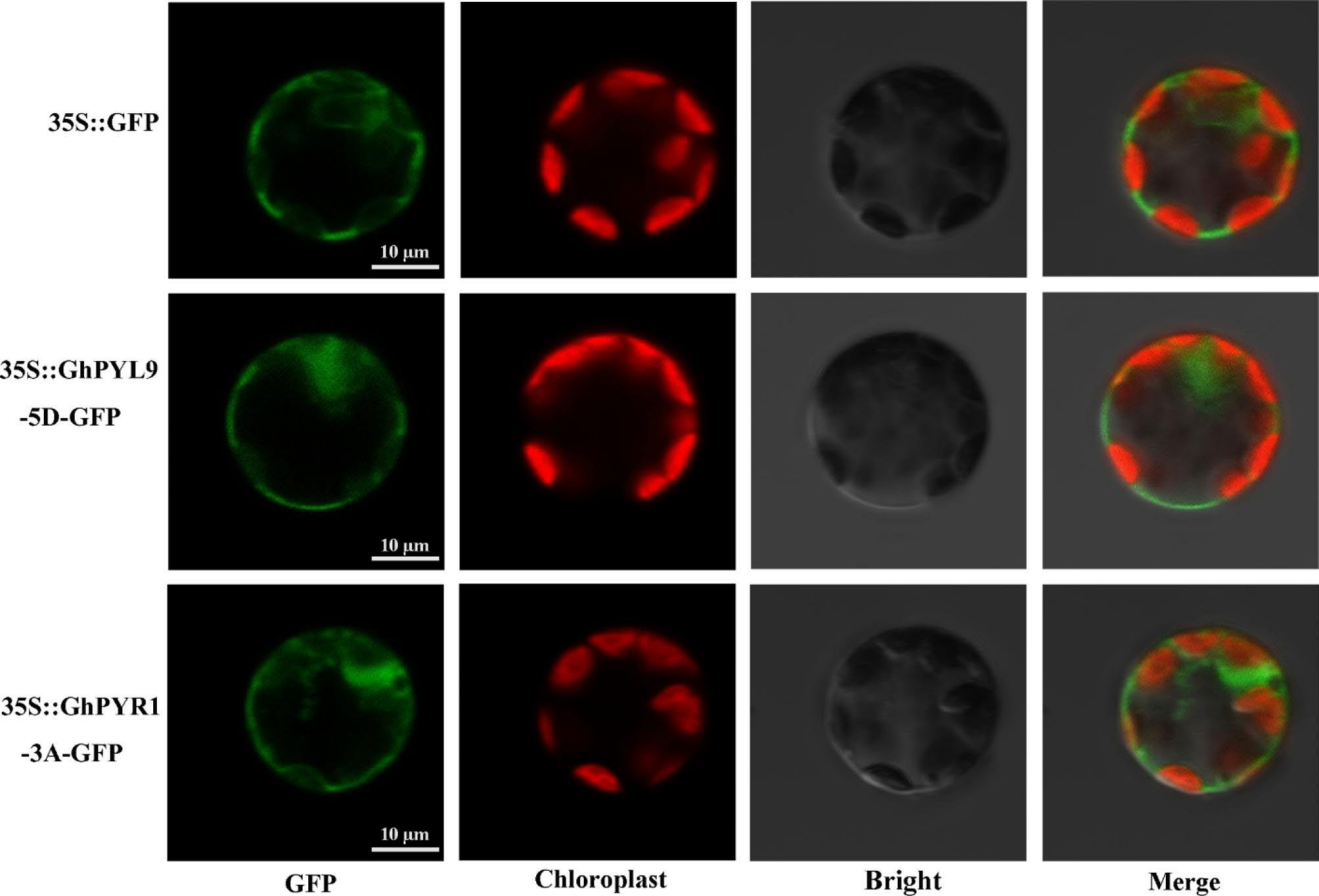
Subcellular localization of GhPYL9-5D and GhPYR1-3A. Vectors of *35S::GFP* (Control), *35S::GhPYL9-5D-GFP* and *35S::GhPYR1-3A-GFP* were introduced into Arabidopsis protoplasts, respectively. GFP signal (GFP) was assayed by a laser confocal-scanning microscope. Red autofluorescence (Chloroplast) is a chloroplast marker, and the “merge” shows an overlap picture of GFP and chloroplast fluorescence. The bar is 10 μm

Previously, we analyzed the structure and phylogenesis of PYL family members in *Gossypium*, and identified 40 GhPYLs including GhPYL9-5D and GhPYR1-3A [40]. In this report, the functions of GhPYL9-5D and GhPYR1-3A were studied, and the subcellular localization of the two proteins was examined. The coding sequences of *GhPYL9-5D* and *GhPYR1-3A* were amplified, respectively, and were fused to the N-terminal region of a GFP fragment driven by 35S promoter in the vector pCAMBIA1300-GFP. The constructed vectors and the corresponding empty vector (Control vector) were then individually introduced into *Arabidopsis* protoplasts. Gene transient expression results revealed that the GFP fluorescence of the two fusion proteins spread in the cytoplasmic and nuclear regions (Fig. 1), indicating that both GhPYL9-5D and GhPYR1-3A are localized in the cytoplasm and nucleus.

### Generation of transgenic lines overexpressing *GhPYL9-5D* and *GhPYR1-3A* in *Arabidopsis* WT and PYL sextuple mutant *112,458*

**Fig. 2:**
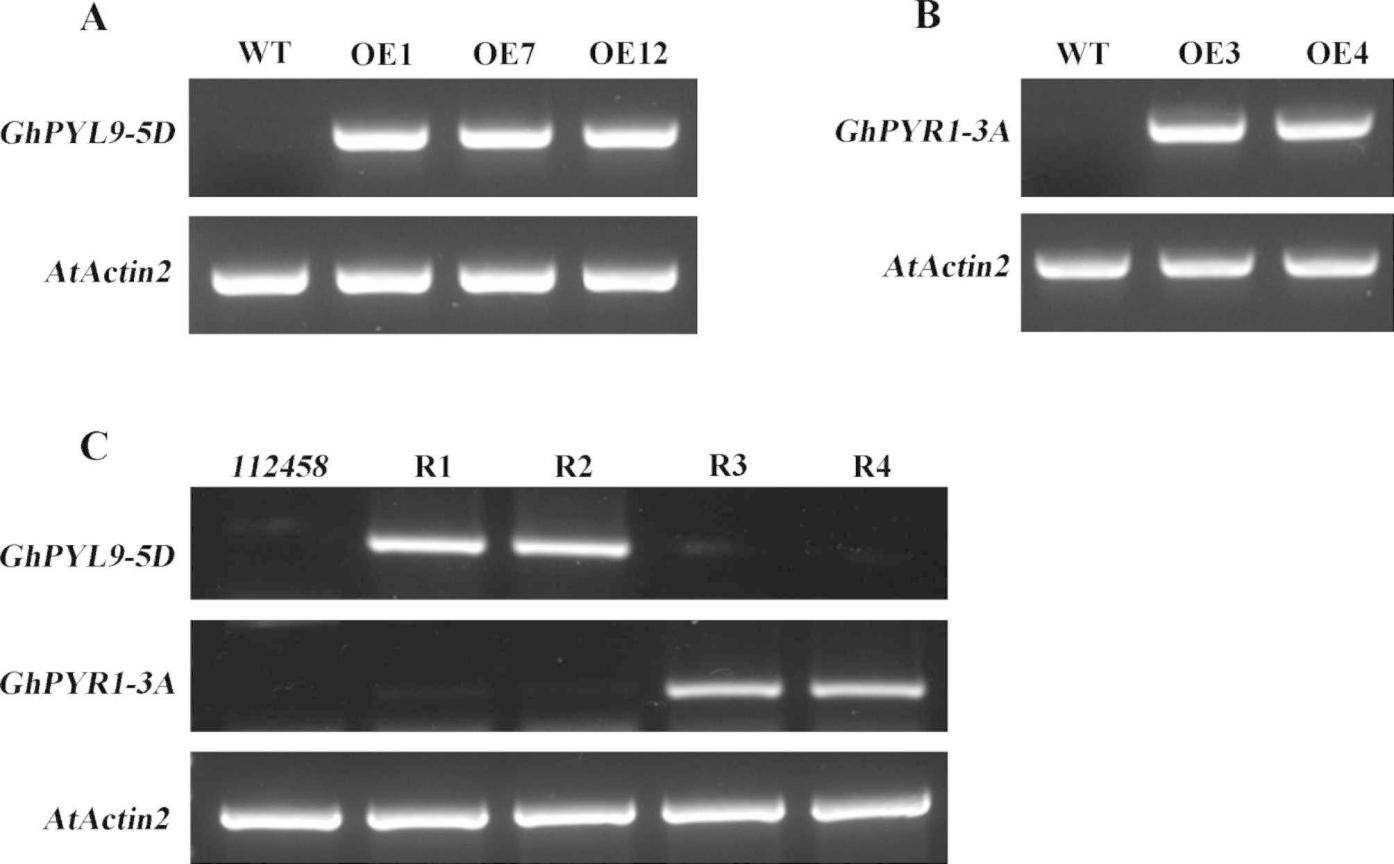
Expression levels of *GhPYL9-5D* and *GhPYR1-3A* in different Arabidopsis transgenic plants. RT-PCR experiments were performed. (**A**) and (**B**) Transcriptional profiles of *GhPYL9-5D* in three lines overexpressing *GhPYL9-5D* in Arabidopsis WT plants and *GhPYR1-3A* in two lines overexpressing *GhPYR1-3A* in Arabidopsis WT plants, respectively. (**C**) Expression of *GhPYL9-5D* and *GhPYR1-3A* in the lines overexpressing *GhPYL9-5D* or *GhPYR1-3A* in *112,458*. The two genes and an internal control *AtActin2* were amplified by 22 cycles in all experiments. The original image was put in the supplementary Fig. 1

To explore the roles of *GhPYL9-5D* and *GhPYR1-3A* in acclimation to stresses, transgenic plants overexpressing each of the two genes were generated. The encoding sequences of *GhPYL9-5D* and *GhPYR1-3A* were respectively cloned, fused with the expression vector pCAMBIA1300 driven by 35S promoter, and introduced into *Arabidopsis* WT and *pyr1pylpyl2pyl4pyl5pyl8* sextuple mutant (abbreviated as *112,458*) plants [14, 43]. Multiple T3 transgenic lines were obtained. RT-PCR results revealed that *GhPYL9-5D* mRNAs were abundant in the lines OE1, OE7 and OE12 (overexpression lines of *GhPYL9-5D* in WT), and in the lines R1 and R2 (recovery line 1 and 2, overexpression lines of *GhPYL9-5D* in *112,458*) (Fig. 2A, C, Fig. S1), and *GhPYR1-3A* expression levels were high in the lines OE3 and OE4 (overexpression lines of *GhPYR1-3A* in WT), and in the line R3 and R4 (overexpression lines of *GhPYR1-3A* in *112,458*) (Fig. 2B, C, Fig. S1).

### Transgenic plants overexpressing *GhPYL9-5D* and *GhPYR1-3A* in *Arabidopsis* WT and *112,458* were sensitive to ABA in term of seed germination

**Fig. 3:**
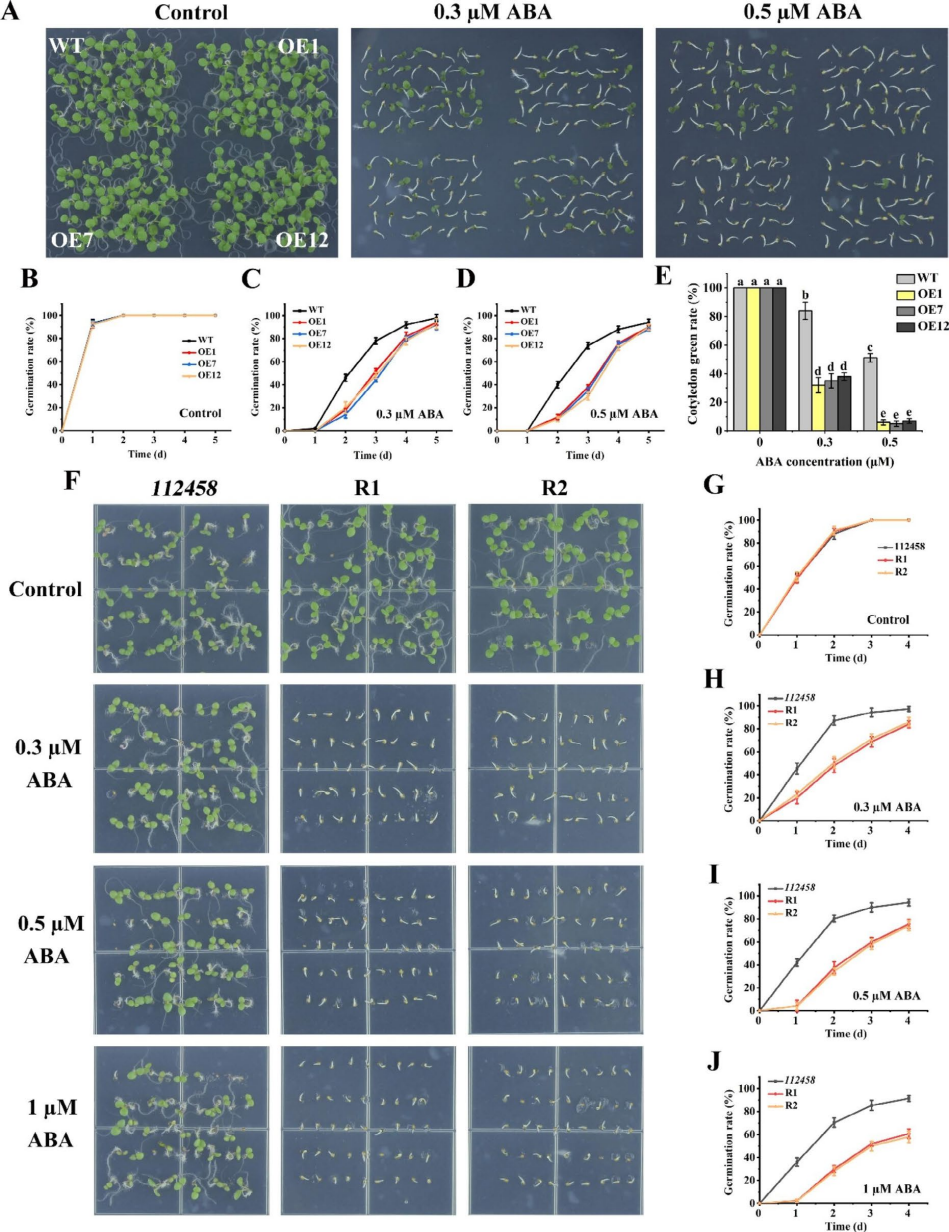
Effects of ABA on the seed germination and cotyledon growth of various plants. (**A**) Seeds of WT, OE1, OE7 and OE12 germinated in MS medium containing 0 (Control), 0.3 or 0.5 µM ABA for 10 d. (**B**)-(**D**) Seed germination rates of WT and *GhPYL9-5D* OE lines in MS medium with 0 (Control), 0.3 and 0.5 µM ABA, respectively. (**E**) Cotyledon green rates of WT and three OE lines in the absence or presence of ABA for 10 d. (**F**) Seeds of *112,458* mutant, and two recovery lines R1 and R2 germinated in MS medium supplied with 0 (Control), 0.3, 0.5 or 1 µM ABA for 7 d. (**G**)-(**J**) Seed germination rates of WT, R1 and R2 in MS medium containing 0 (Control), 0.3, 0.5 and 1 µM ABA, respectively. Data are mean ± SD (n ≥ 3). Different lowercase letters above the bars represent significant differences between two lines by one way ANOVA and Tukey’s HSD test (*P* < 0.05)

**Fig. 4:**
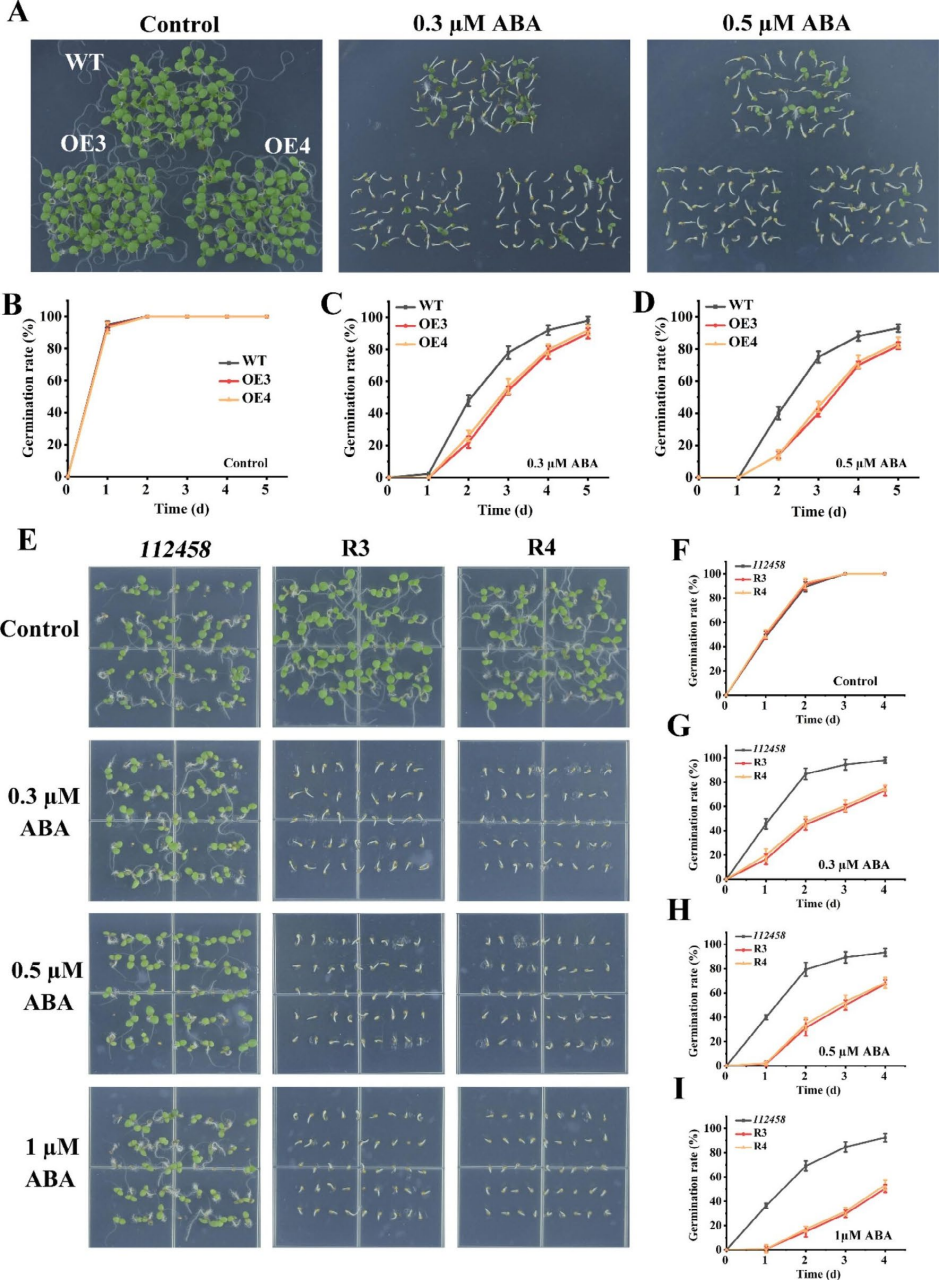
Effects of ABA on seed germination of different plants. (**A**) Seeds of WT, OE3 and OE4 germinated in MS medium containing 0 (Control), 0.3 or 0.5 µM ABA for 10 d. (**B**)-(**D**) Seed germination rates of WT and the *GhPYR1-3A* OE lines in MS medium supplied with 0 (Control), 0.3 and 0.5 µM ABA, respectively. (**E**) Seeds of *112,458* mutant and the two recovery lines R3 and R4 germinated in MS medium with 0 (Control), 0.3, 0.5 or 1 µM ABA for 7 d. (F)-(I) Seed germination rates of WT, R3 and R4 in MS medium supplemented with 0 (Control), 0.3, 0.5 and 1 µM ABA, respectively. Data are mean ± SD (n ≥ 3)

To clarify whether GhPYL9-5D and GhPYR1-3A modulate ABA-mediated seed germination, the germination performances of their overexpression lines and WT seeds were compared. In Murashige-Skoog (MS) medium, the seed germination percentages of all plants were similar (Figs. 3A and B and 4A and B). However, in MS medium supplemented with 0.3 or 0.5 µM ABA for 2 and 3 d, the seeds of OE1, OE7 and OE12 germinated clearly slower than those of WT. After treatment with 0.3 and 0.5 µM ABA for 3 d, the average seed germination rates of the three OE lines were 48% and 34%, respectively, whereas those of WT were 78% and 74%, respectively. Cotyledon green rates of the three OE lines were also significantly lower than those of WT in the presence of 0.3 and 0.5 µM ABA (Fig. 3A-E). Similarly, *GhPYR1-3A* transgenic lines OE3 and OE4 displayed notably lower seed germination rates than WT after grown in MS medium containing 0.3 or 0.5 µM ABA for 2 and 3 d (Fig. 4A-D). These results imply that GhPYL9-5D and GhPYR1-3A exert positive effects on ABA-suppressed seed germination.

To ascertain whether GhPYL9-5D and GhPYR1-3A have similar functions to *Arabidopsis* ABA receptors, the impacts of ABA on seed germination were studied in *112,458*, R1, R2, R3 and R4. As expected, *112,458* exhibited extremely high insensitivity to ABA in term of seed germination. By contrast, R1, R2, R3 and R4 were very sensitive to 0.3, 0.5 and 1 µM ABA during seed germination. Moreover, the inhibitory effects of ABA on seed germination of these transgenic lines were dose-dependent (Figs. 3F-J and 4E-I), indicating that GhPYL9-5D and GhPYR1-3A may act as ABA receptors in the regulation of ABA-mediated seed germination.

### Overexpression of *GhPYR1-3A* but not *GhPYL9-5D* in *Arabidopsis* WT conferred seed germination hypersensitivity to salt and osmotic stress

To understand whether GhPYL9-5D and GhPYR1-3A function in responding to salt and osmotic stress, the effects of NaCl and mannitol on seed germination and cotyledon growth of their overexpression lines and WT were determined. Surprisingly, no significant differences in seed germination rates and cotyledon green rates were observed between the *GhPYL9-5D* transgenic plants OE1, OE7, OE12 and WT in MS medium supplied without or with 100 and 120 mM NaCl, 200 and 250 mM mannitol (Fig. S2). By contrast, *GhPYR1-3A* overexpression lines OE3 and OE4 were noticeably more sensitive to 75 and 100 mM NaCl, and to 200 and 250 mM mannitol than WT during seed germination. The two OE lines had no significant differences in the seed germination rates from WT under the control conditions (Fig. S3). These data suggest that high levels of *GhPYR1-3A* but not of *GhPYL9-5D* cause the inhibitory effects on seed germination of *Arabidopsis* WT plants under salt and osmotic stress.

### Expression of both *GhPYL9-5D* and *GhPYR1-3A* attenuated the insensitivity of *112,458* to saline and osmotic stress in seed germination

To define whether GhPYL9-5D and GhPYR1-3A play a similar part to *Arabidopsis* ABA receptors in response to salinity and osmotic stress, seed germination of *112,458*, R1, R2, R3 and R4 was analyzed. The results showed that R1, R2, R3 and R4 had marked reduction in seed germination rates after grown upon 100 mM NaCl, 200 mM mannitol, and 250 mM mannitol for 2 and 3 d in comparison with *112,458*. In contrast, the seeds of these transgenic lines germinated as faster as *112,458* under the control conditions (Fig. S4, S5). These results demonstrate that the expression of *GhPYL9-5D* or *GhPYR1-3A* can rescue the insensitivity phenotypes of *112,458* mutant plants to salinity and osmotic stress during seed germination.

### *GhPYL9-5D* expression in *Arabidopsis* WT plants strengthened ABA- rather than NaCl- and mannitol-inhibited primary root growth

**Fig. 5:**
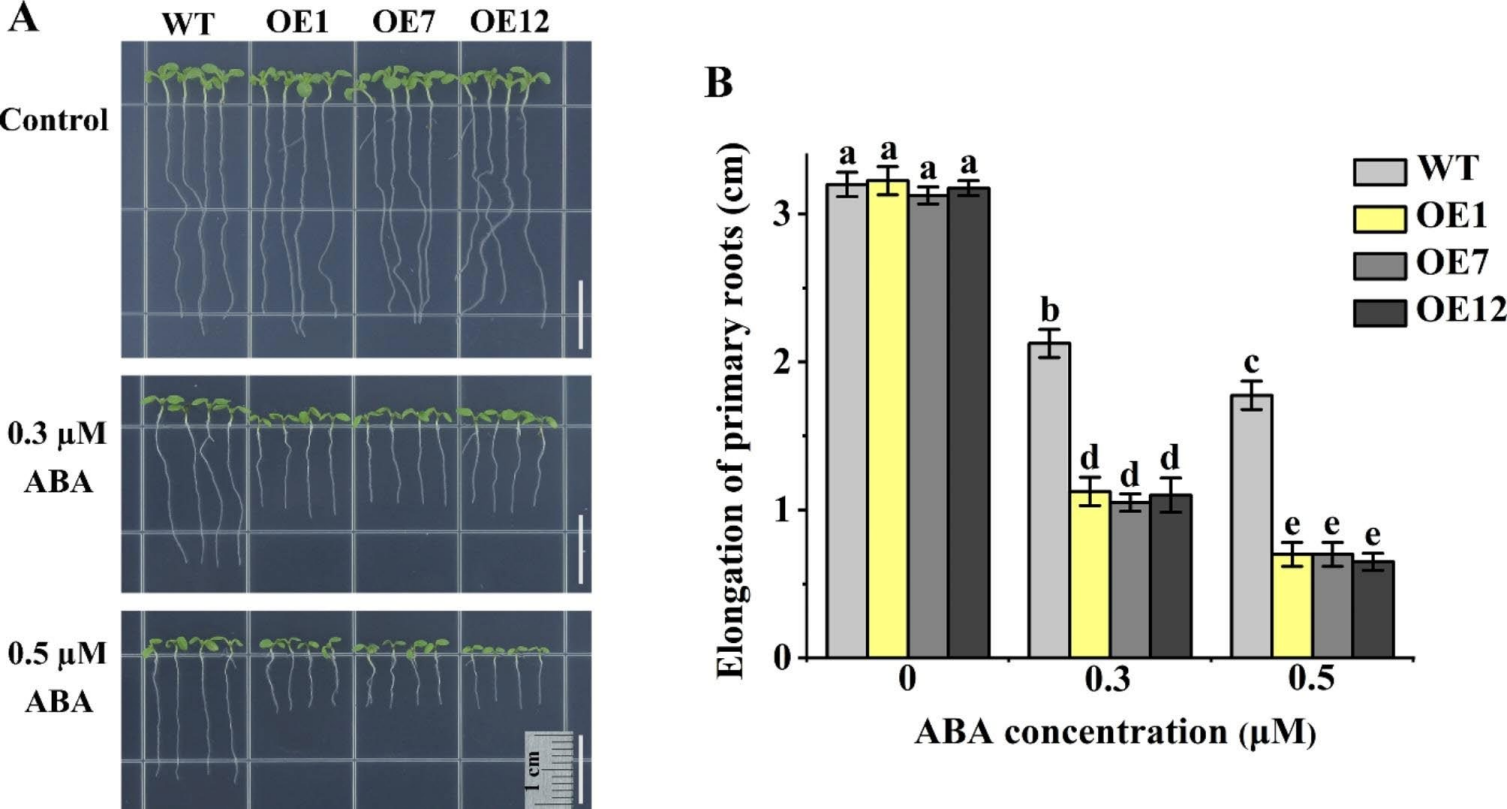
Root growth of transgenic Arabidopsis plants overexpressing *GhPYL9-5D* was sensitive to ABA. (**A**) Growth performances of OE1 OE7, OE12 and WT seedlings. Bar is 1 cm. (**B**) Enhancements of primary root length of various plants. Three day-old seedlings were transferred to MS medium supplied with 0 (Control), 0.3 or 0.5 µM ABA for 6 d. Data are mean ± SD (n ≥ 30). Different lowercase letters above the error bars reveal significant differences between two lines by one way ANOVA and Tukey’s HSD test (*P* < 0.05)

To gain insight into the functions of GhPYL9-5D in root growth, young seedlings of OE1, OE7, OE12 and WT were transferred to MS medium without or with ABA (0.3 and 0.5 µM), NaCl (100 and 120 mM) and mannitol (200 and 250 mM), and grown for a period of time. In the absence of ABA, the elongation of primary roots of all plants was similar. However, in the presence of 0.3 and 0.5 µM ABA, root growth of the three OE lines was remarkably slower than that of WT (Fig. 5). Interestingly, the three OE lines showed no significant differences from WT in the enhanced length of primary roots in MS medium containing 0, 100 or 120 mM NaCl. The increments in primary root length of OE1, OE7 and OE12 were slightly greater than WT after treatment with 200 mM mannitol, and were clearly greater than WT after treatment with 250 mM mannitol (Fig. S6). The results above signify that GhPYL9-5D fulfills positive regulatory functions in ABA-inhibited primary root elongation, and in seedling tolerance to salt and osmotic stress in *Arabidopsis*.

### *GhPYR1-3A* overexpression markedly enhanced the root growth sensitivity of *Arabidopsis* WT seedlings to ABA and mannitol but not to NaCl

**Fig. 6:**
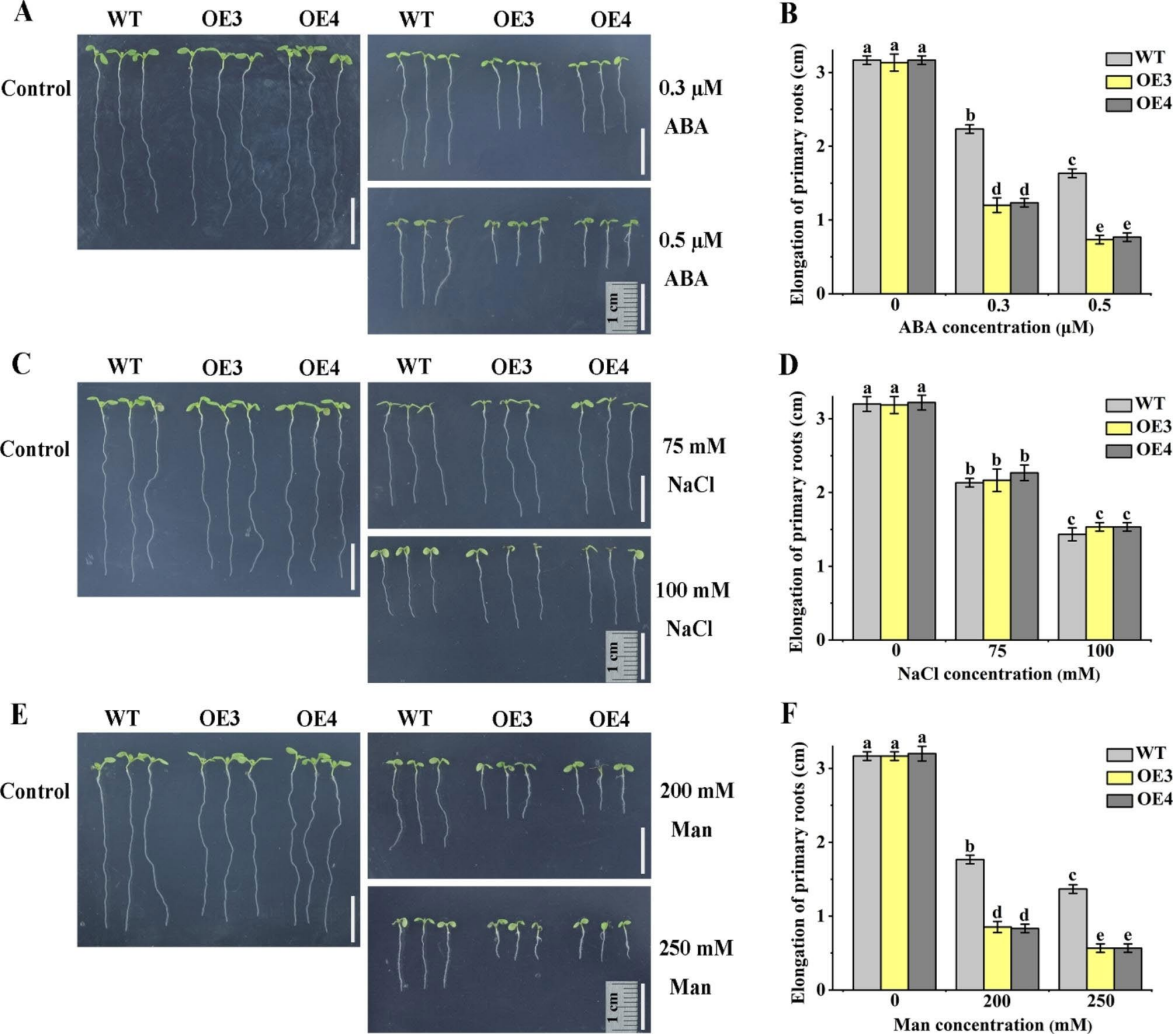
*GhPYR1-3A* overexpression lines showed high sensitivity to ABA and osmotic stress. (**A**), (**C**) and (**E**) Growth performances of OE3 and OE4 and WT plants. Bar is 1 cm. (**B**), (**D**) and (**F**) Primary root elongation of various lines. Three-day-old seedlings were transferred to MS medium not containing (Control) or containing different concentrations of ABA (0.3 and 0.5 µM), NaCl (75 and 100 mM) or mannitol (Man, 200 and 250 mM) for 6 d. Data are mean ± SD (n ≥ 30). Different lowercase letters above the error bars show marked differences between two seedlings by one way ANOVA and Tukey’s HSD test (*P* < 0.05)

We investigated the roles of GhPYR1-3A in *Arabidopsis* root growth, and found that the root elongation of *GhPYR1-3A* overexpression lines OE3 and OE4 was remarkable smaller than that of WT in MS medium containing ABA (0.3 and 0.5 µM), or mannitol (200 and 250 mM). In contrast, the increases in root length of OE3 and OE4 were similar to those of WT in MS medium (Fig. 6A, B, E, F). No significant differences in root elongation were observed between the two OE lines and WT in the absence and presence of NaCl (75 and 100 mM) (Fig. 6C, D). Our findings imply that GhPYR1-3A has repressed effects on primary root growth in responding to ABA and osmotic stress but not to salinity in *Arabidopsis*.

### Root growth of *GhPYL9-5D* and *GhPYR1-3A* overexpression lines in *112,458* was sensitive to ABA, but insensitive to NaCl and mannitol

**Fig. 7:**
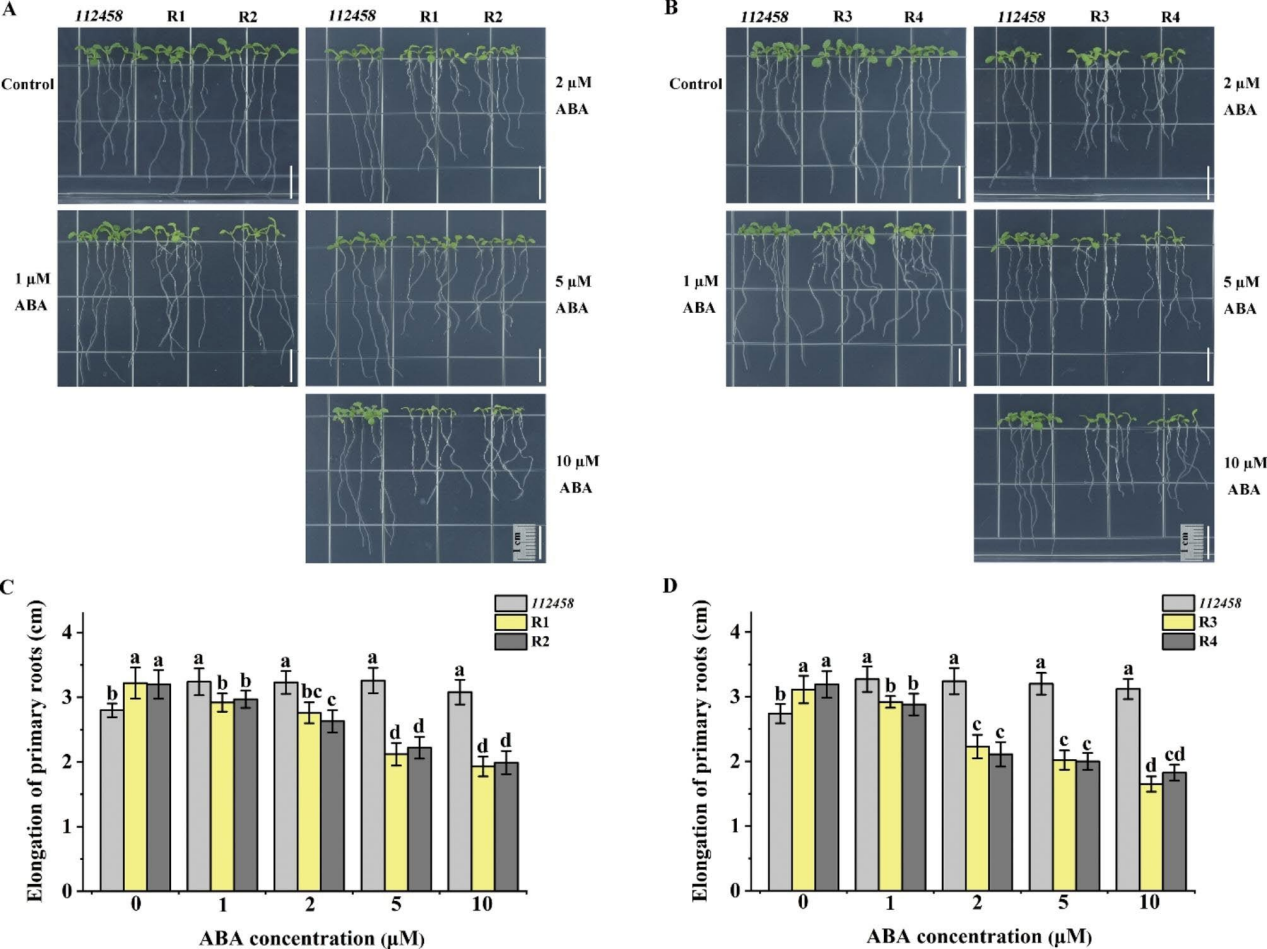
Both GhPYL9-5D and GhPYR1-3A alleviated the insensitive effects of *112,458* to ABA in term of root elongation. (**A**) and (**B**) Seedling growth performances of *112,458* mutant, R1, R2, R3 and R4. Bar is 1 cm. (**C**) and (**D**) Increased length of primary roots of various plants. Three-day-old seedlings were transferred to MS medium supplemented with 0 (Control), 1, 2, 5 or 10 µM ABA for 7 d. Data are mean ± SD (n ≥ 30). Different lowercase letters above the error bars indicate significant differences between two lines by one way ANOVA and Tukey’s HSD test (*P* < 0.05)

To determine whether GhPYL9-5D and GhPYR1-3A act as ABA receptors in *Arabidopsis*, root elongation of R1, R2, R3, R4 and *112,458* was assayed. The results revealed that the increments in primary root length of all plants were similar in MS medium; however, those of R1, R2, R3 and R4 were evidently smaller than those of *112,458* in MS medium containing 2, 5 or 10 µM ABA. Moreover, the effects of ABA on root elongation of these plants depended on its concentrations (Fig. 7), pointing to the important roles of GhPYL9-5D and GhPYR1-3A in rescuing the deficiency of *PYR1*, *PYL1*, *PYL2*, *PYL4*, *PYL5* and *PYL8* in *Arabidopsis* response to ABA.

Interestingly, the roots of R1, R2, R3 and R4 elongated markedly longer than those of *112,458* in MS medium containing 75 and 100 mM NaCl, or 200 and 250 mM mannitol. In contrast, the root growth of all the four transgenic lines was similar to that of *112,458* in MS medium (Fig. S7), suggesting that both GhPYL9-5D and GhPYR1-3A might function as ABA receptors in the modulation of primary root growth in response to ABA, and in seedling tolerance to salt and osmotic stress in *Arabidopsis*.

### Transgenic plants overexpressing *GhPYL9-5D* and *GhPYR1-3A* in *112,458* showed significantly improved tolerance to drought and salinity

**Fig. 8:**
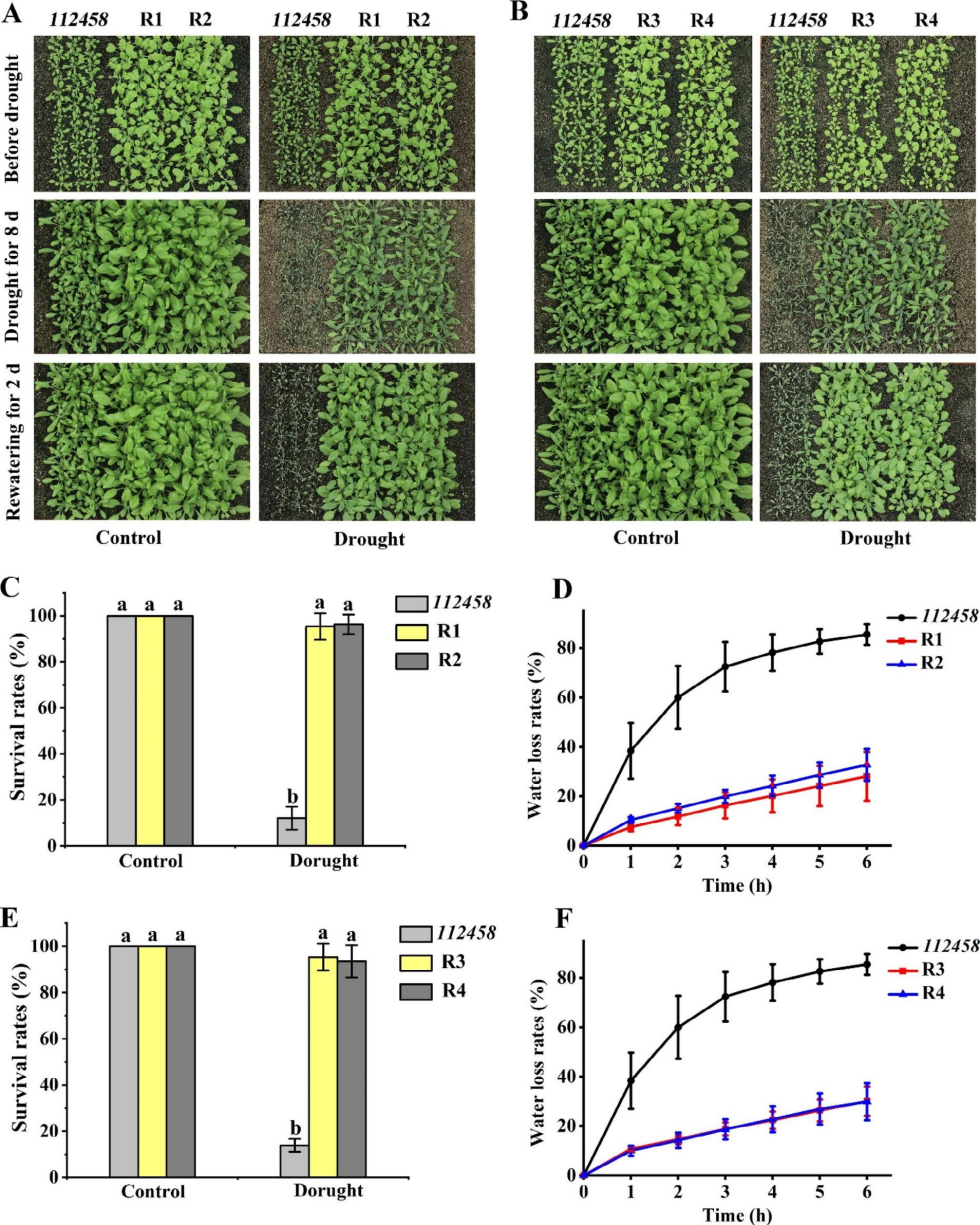
*GhPYL9-5D* and *GhPYR1-3A* overexpression plants of *112,458* displayed increased tolerance to water deficit. (**A**) Growth performances of *112,458* mutant, R1 and R2. (**B**) Growth performances of *112,458*, R3 and R4. (**C**) and (**E**) Survival rates of various plants. (**D**) and (**F**) Water loss rates of different lines. Four-week-old plants were subjected to drought stress by withholding water for 8 d. Then, the plants were rewatered for 2 d. Survival rates of all plants were evaluated after rewatering. Water loss rates of 4-week-old plants were assayed. Data are mean ± SD (n ≥ 3). Different lowercase letters above the error bars reveal significant differences between two lines by one way ANOVA and Tukey’s HSD test (*P* < 0.05)

We next investigated the roles of GhPYL9-5D and GhPYR1-3A in *Arabidopsis* adaptation to dehydration and salt stress in soil. It was observed that four-week-old plants of R1, R2, R3 and R4 grew apparently better than *112,458* under normal growth conditions. After grown for 8 d without irrigation, most of *112,458* seedlings wilted and died whereas the majority of the four transgenic lines did not wilt or only slightly wilted, and were green. After rewatering for 2 d, nearly all of the *112,458* plants failed to survive. By contrasts, the transgenic plants grew normally. In addition, the survival rates of R1, R2, R3 and R4 were remarkably higher than those of WT, and leaf water loss rates of the four transgenic lines were dramatically lower than those of WT (Fig. 8). Our findings support the notion that GhPYL9-5D and GhPYR1-3A confer drought tolerance in *Arabidopsis 11,248* mutant.

In the absence of NaCl, R1, R2, R3 and R4 grew pronouncedly faster than *112,458*. After treatment with 200 and 250 mM NaCl for 14 d, the growth of all plants was markedly suppressed. It was noteworthy that the four transgenic lines exhibited clearly less serious growth inhibition than *112,458* plants. The survival rates of the transgenic plants were also significantly higher than those of *112,458* in the presence of NaCl (Fig. S8). These results demonstrate that overexpression of *GhPYL9-5D* and *GhPYR1-3A* effectively rescues the growth defect of *112,458* mutant plants under saline conditions.

### ABA induced stomatal closure in the transgenic lines overexpressing *GhPYL9-5D* and *GhPYR1-3A* in *112,458*

**Fig. 9:**
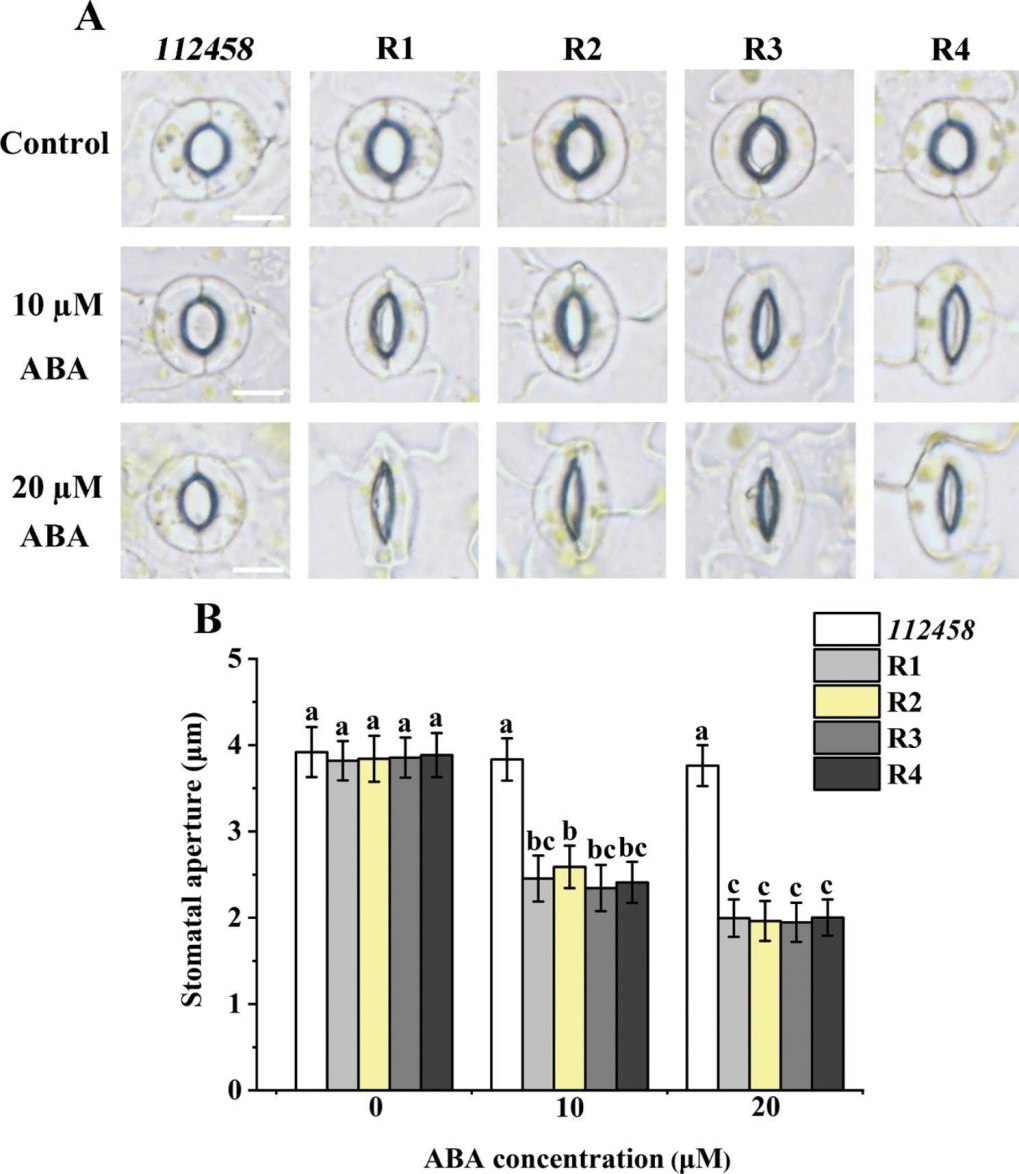
Expression of *GhPYL9-5D* and *GhPYR1-3A* in *112,458* casued significant increases in the sensitivity of transgenic plants to ABA-induced stomatal closure. (**A**) Microscopy images for ABA-induced stomatal closure. (**B**) Stomatal aperture of *112,458*, R1, R2, R3 and R4 in the absence and presence of 10 or 20 µM ABA. Abaxial epidermal strips were incubated in buffer solution in light for 2.5 h to open stomata, and then were subjected to 10 or 20 µM ABA for another 0.5 h. Values are means ± SD (n ≥ 50). Different lowercase letters indicate values from the seedlings significantly differ from each other by one way ANOVA and Tukey’s HSD test (*P* < 0.05). Bar is 5 μm

To make certain whether the expression of *GhPYL9-5D* and *GhPYR1-3A* in *112,458* influences stomatal closure, changes in stomatal aperture in *112,458*, R1, R2, R3 and R4 after ABA treatment were assessed. The results showed that *112,458* was insensitive to ABA-elicited stomatal closure. Minor decrease in stomatal aperture upon 10 and 20 µM ABA was observed in *112,458*. By contrast, the stomatal aperture of the four transgenic lines was clearly reduced after treatment with the same concentrations of ABA (Fig. 9), indicating that GhPYL9-5D and GhPYR1-3A may act as ABA receptors to positively modulate ABA-triggered stomatal closure.

### Silencing of *GhPYL9-5D* and *GhPYR1-3A* evidently decreased cotton tolerance to PEG-induced drought, salinity and osmotic stress

**Fig. 10:**
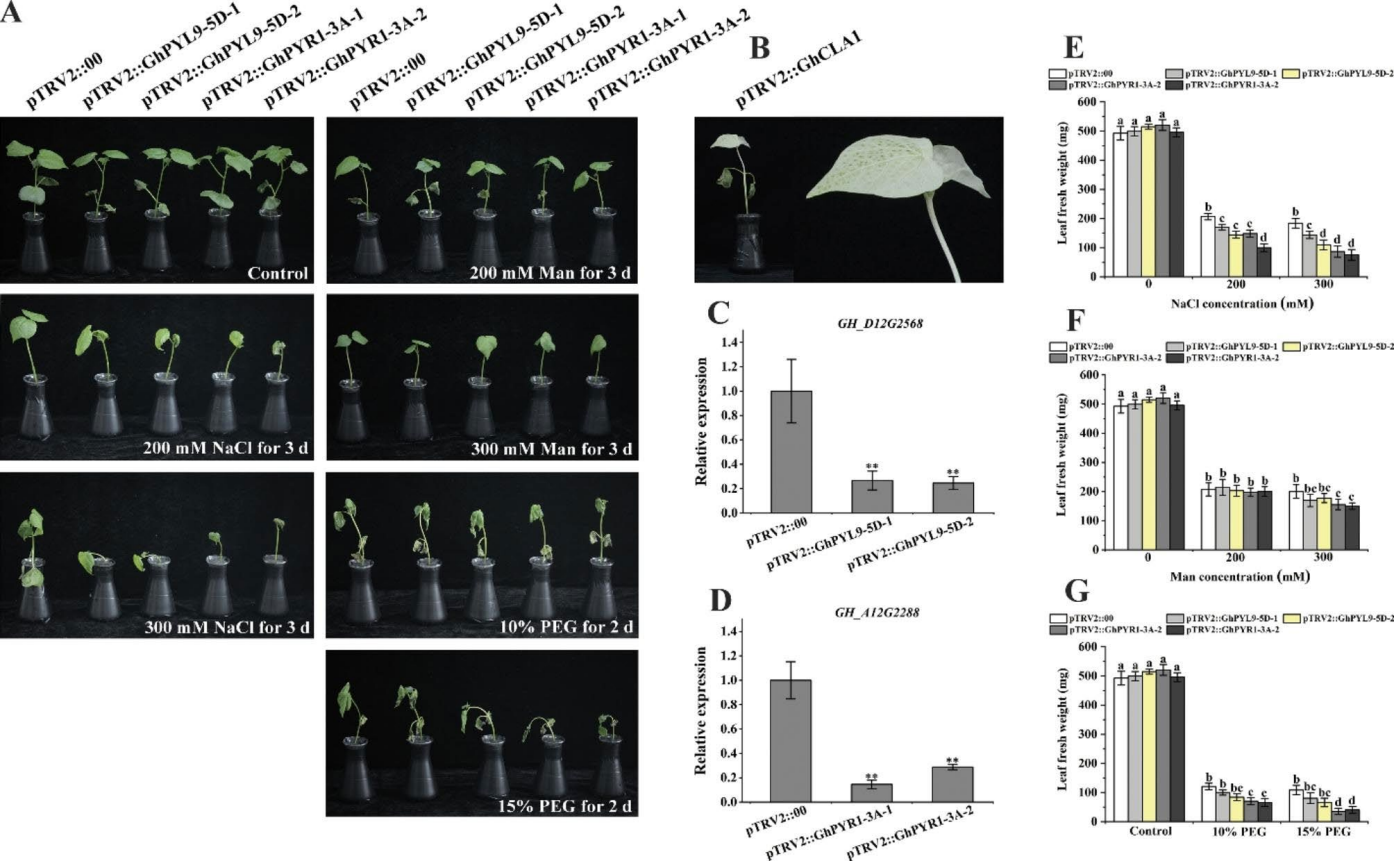
Silencing *GhPYL9-5D* and *GhPYR1-3A* in cotton reduced plant tolerance to PEG-triggered drought, salinity and osmotic stress. (**A**) Growth performances of various VIGS plants. (**B**) Albino phenotype of *pTRV2::GhCLA1* plants. (**C**) and (**D**) Relative expression of *GhPYL9-5D* and *GhPYR1-3A*, respectively, in their corresponding VIGS lines. (**E**)-(**G**) Leaf fresh weights of different lines after challenge with PEG, NaCl and mannitol (Man), respectively. One-week-old seedlings grown on vermiculite irrigated with Hoagland’s nutrient solution were infected by VIGS solutions. One week later, the plants were subjected to PEG, NaCl and mannitol stress for indicated days, and leaf fresh weight were assayed. Expression levels of *GhPYL9-5D* and *GhPYR1-3A* were measured before stress treatment. Values are means ± SD (n ≥ 3). The double asterisk represents that the data from VIGS plants of *GhPYL9-5D* and *GhPYR1-3A* significantly differ from the control by student’s *t* test (*P* ≤ 0.01). Different lowercase letters above the error bar reveal that the values between two VIGS plants are significantly different by one-way ANOVA and Tukey’s HSD test (*P* ≤ 0.05)

To further explore the functions of *GhPYL9-5D* and *GhPYR1-3A* in cotton, virus-induced gene silencing (VIGS) methods were applied to silence the two genes. Cotton plants infiltrated with *Agrobacterium* cultures containing the vectors *pTRV2::GhCLA1* (*Cloroplastos alterados 1*, positive control), *pTRV2::GhPYL9-5D*, *pTRV2::GhPYR1-3A* and *TRV2::00* (empty vector, negative control) were obtained. Leaves of the seedlings harboring *pTRV2::GhCLA1* displayed the albino phenotype, and the expression levels of *GhPYL9-5D* and *GhPYR1-3A* were significantly reduced in their corresponding VIGS lines. After grown in Hoagland’s nutrient solution (the control condition) for 3 d, the *pTRV2::GhPYL9-5D* and *pTRV2::GhPYR1-3A* lines displayed the similar growth performances to *TRV2::00* plants. However, in Hoagland’s nutrient solution supplied with polyethylene glycol 6000 (PEG, 10% and 15%) for 2 d, with NaCl (200 and 300 mM) for 3 d or with mannitol (200 and 300 mM) for 3 d, *pTRV2::GhPYL9-5D* and *pTRV2::GhPYR1-3A* plants grew apparently worse than the *pTRV2::00* line (Fig. 10A-D). Although the leaves of all plants wilted after challenge with PEG, the degree of leaf wilting of *pTRV2::GhPYL9-5D* and *pTRV2::GhPYR1-3A* seedlings was observably higher than that of *pTRV2::00* plants. After treatment with 200 mM NaCl, all of the leaves of *pTRV2::GhPYL9-5D* and *pTRV2::GhPYR1-3A* plants wilted. In contrast, the top two leaves of *pTRV2::00* lines were not wilted. Upon 300 mM mannitol treatment, the true leaves from *pTRV2::GhPYL9-5D* and *pTRV2::GhPYR1-3A* plants grew downward. By contrast, those from *TRV2::00* plants were roughly horizontal (Fig. 10A). The leaf fresh weights of *pTRV2::GhPYL9-5D* and *pTRV2::GhPYR1-3A* seedlings were also significantly smaller than those of *pTRV2::00* plants after treatment with PEG, NaCl and mannitol (Fig. 10E-G). Together, these results indicate that GhPYL9-5D and GhPYR1-3A are two positive regulators of cotton tolerance to drought, high salinity and osmotic stress.

### *GhPYL9-5D*, *GhPYR1-3 A* and their homologs were strongly expressed under abiotic stresses as well as in roots in cotton

To further explore the potential roles of *GhPYL9-5D* and *GhPYR1-3A* in cotton response to abiotic stress including drought and high salinity, the expression patterns of the two genes and their homologs were analyzed using the published transcriptomic data. Expression information of 14 *GhPYL9* genes and 6 *GhPYR1* genes was obtained (Fig. S9). It was found that the transcriptional levels of *GhPYL9-5D* and *GhPYR1-3A* remarkably increased after treatment with 20% PEG for 24 h. Moreover, the expression profiles of *GhPYL9-2D*, *GhPYL9-7D* and *GhPYL9-1A* were similar to *GhPYL9-5D* after exposure to PEG for 24 h (Fig. S9A). The expression of *GhPYL9-5D* and *GhPYR1-3A* was clearly upregulated by 400 mM NaCl treatment for 1 and 24 h, respectively (Fig. S9B). The expression of *GhPYL9-5D* and *GhPYR1-3A* markedly enhanced after exposure of cotton plants to 37℃ for 3 and 24 h, respectively (Fig. S9C). Both *GhPYL9-5D* and *GhPYR1-3A* showed strong expression under low temperature stress (4℃) for 1 h (Fig. S9D). Additionally, the transcription of multiple homologous members of *GhPYL9-5D* and *GhPYR1-3A* was induced by the above stresses. These results suggest that GhPYL9s and GhPYR1s may be of general importance in cotton responding to drought, salt, high and low temperature stresses.

The mRNA abundances of these *GhPYL9s* and *GhPYR1s* in various tissues were analyzed using published transcriptome data. Six *GhPYL9s* including *GhPYL9-5D* were highly expressed in the root, and *GhPYR1-3A* was strongly expressed in the fiber and stem (Fig. S9E), reflecting the distinct roles of GhPYL9 homologs and GhPYR1-3A in diverse tissues of cotton. To understand the signal transduction processes modulated by GhPYL9s and GhPYR1s, the promoter sequences of 14 *GhPYL9s* and 6 *GhPYR1s* were analyzed. The predicted cis-elements were related to the processes of drought inducibility, ABA signaling, light signaling, auxin responsiveness, low temperature response, and stress responsiveness. Of note, ABA responsive elements existed in the promotor regions of most *GhPYL9* and *GhPYR1* members (Fig. S9F, G). These data hint that GhPYL9s and GhPYR1s including GhPYL9-5D and GhPYR1-3A may play essential roles in multiple aspects of growth and development and in response to diverse environmental stimuli, especially in ABA signaling in cotton.

### Numerous genes were co-expressed with *GhPYL9-5D* and *GhPYR1-3 A* in response to drought or saline stress

**Fig. 11:**
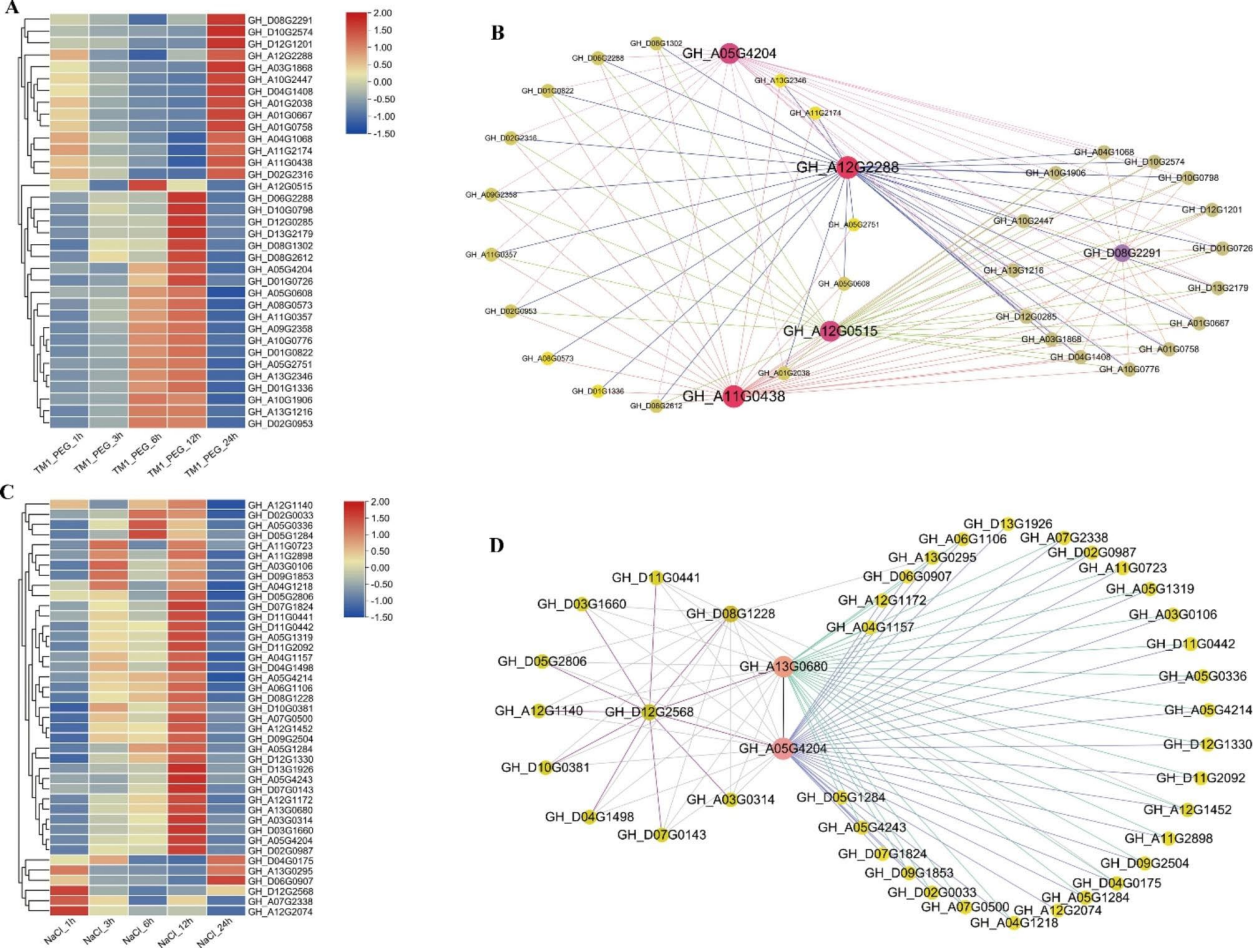
Heatmap and co-expression networks of DEGs in cotton response to PEG or NaCl treatment (**A**) Heatmap plots of 35 DEGs after 20% PEG treatment for indicated hours. (**C**) Heatmap plots of 41 DEGs after treatment with 400 mM NaCl for indicated hours. The red represents that gene expression is upregulated, and the blue indicates that gene expression is downregulated. (**B**) and (**D**) Gene co-expression networks for the 35 DEGs and 41 DEGs, respectively. Nodes represent genes. The red nodes mean hub genes

To gain insight into the potential genes working in coordination with *GhPYL9-5D* and *GhPYR1-3A* in responding to drought or salt stress, co-expression networks were constructed using the two genes as hub genes, and applying the differential expressed genes (DEGs) from the transcriptomic data of cotton L. acc. Texas Marker-1 (TM-1) plants. Totally, 35 DEGs after 20% PEG treatments and 41 DEGs after challenge with 400 mM NaCl were implicated in the expression modules affected by *GhPYL9-5D* and *GhPYR1-3A*, respectively (Fig. 11). The transcription of 34 out of 35 DEGs was upregulated after exposure to PEG for 12 or 24 h. In addition, a number of genes including GH_A05G4204 (*NADPH quinone oxidoreductase*, *NQR*), GH_A11G0438 (*PP2C60*), GH_A12G0515 (*Suppressor of ABA insensitive 3-5*), and GH_D08G2291 (*PP2C25*) were interconnected with *GhPYL9-5D* (Fig. 11A, B), hinting that GhPYL9-5D may regulate cotton response to drought stress via interacting with the components of ABA signaling and ROS signaling. Upon high salinity for 12 h, the expression levels of the majority of the 41 genes enhanced. *GhPYR1-3A* was interconnected with many genes such as GH_A05G4204, GH_A13G0680 (*Calcium-dependent protein kinase 4, CPK4*), GH_A05G4243 (*WRKY DNA-binding protein 70*, *WRKY70*), GH_A04G1157 (*WRKY33*), GH_A06G1106 (*WRKY40*), GH_D07G1824 (*WRKY1*), GH_D11G0441 (*Ethylene response factor 2*, *ERF2*), GH_A03G0106 (*ERF5*), GH_A11G0723 (*NAC domain containing protein 100*, *NAC100*), GH_D07G0143 (*PP2C77*), GH_D05G1284 (*Auxin resistant 22*, *AUX22*), GH_D09G1853 (*PIN-FORMED 1*, *PIN1*), and GH_A05G1319 (*Indoleacetic acid-induced protein 27*, *IAA27*) in the network (Fig. 11C, D). These data indicate that GhPYL9-5D and GhPYR1-3 A may serve important roles through interplaying with redox signaling components, transcription factors and auxin signal components in cotton adaptation to salt stress.

## Discussion

In the present study, we found that GhPYL9-5D and GhPYR1-3A may function as ABA receptors to positively affect ABA-mediated seed germination, root growth and stomatal closure in *Arabidopsis*. GhPYL9-5D and GhPYR1-3A also play crucial roles in response to drought, high salinity and osmotic stress in *Arabidopsis* and cotton likely through modulating the expression of a number of ABA- or stress-responsive genes.

We observed that cotton GhPYL9-5D and GhPYR1-3A were localized in cytoplasm and nucleus (Fig. 1), being in agreement with the locations of PYLs from *Arabidopsis*, rice, soybean and *Artemisia annua* plants [7, 27, 35, 44]. These results imply that the functions of PYLs may be conserved among cotton and other plant species.

ABA plays essential roles in controlling seed germination and seedling growth in plants. In *Arabidopsis*, nearly all AtPYL members are positive modulators of ABA-inhibited seed germination and seedling growth [14, 15, 19, 21]. Two of these AtPYLs are AtPYL9 and AtPYR1, the homologs of GhPYL9-5D and GhPYR1-3A, respectively, in cotton. In rice, OsPYL9 and OsPYL1, the orthologs of AtPYL9 and AtPYR1, have also been addressed to function redundantly with other OsPYL members in affecting ABA-repressed seed germination [24, 27]. Moreover, OsPYL1 exerts effects redundantly with other OsPYLs in the regulation of ABA-influenced seedling growth [24]. It has been documented that overexpression of *Artemisia AaPYL9*, maize *ZmPYL9*, cotton *GhPYL9-11A*, grape *VyPYL9* and poplar *PePYL6* and *PePYL9* in *Arabidopsis*, and of *SlPYL9* in tomato notably increases ABA sensitivity in terms of seed germination and root growth [28–30, 33, 35]. Down-regulation of *SlPYL9* causes ABA hyposensitivity during seed germination and root growth [29]. Transgenic *Arabidopsis* plants overexpressing poplar *PtPYRL1* and tomato 8g076960, the homologues of *AtPYR1*, display markedly elevated susceptibility to ABA during seed germination or root growth [26, 37]. These results are consistent with our findings about the roles of GhPYL9-5D and GhPYR1-3A in ABA-mediated seed germination and root growth (Figs. 3, 4, 5, 6 and 7), suggesting that PYR1, particularly PYL9, may be of more general importance in ABA-modulated seed germination and root growth in plants.

Transgenic *Arabidopsis* plants overexpressing *GhPYR1-3A* rather than *GhPYL9-5D* showed enhanced sensitivity to high concentrations of NaCl and mannitol during seed germination (Fig. S2, S3). Similar results have been reported in transgenic lines expressing *GhPYL9-11A* in *Arabidopsis* [42], indicating GhPYR1-3 A and GhPYL9-11A but not GhPYL9-5D modulates salt and/or osmotic stress-impacted seed germination in an ABA-dependent pattern. We found that the expression of *GhPYL9-5D* and *GhPYR1-3A* in *112,458* mutant plants led to enhanced seed germination sensitivity to salinity and osmotic stress (Fig. S4, S5), demonstrating that both GhPYL9-5D and GhPYR1-3A are required for salt- and osmotic stress-affected seed germination in the ABA receptor mutant.

The transgenic lines overexpressing *GhPYL9-5D* in *Arabidopsis* exhibited increased root growth upon salt and osmotic stress (Fig. S6). Similar situation was seen in the overexpression lines of *GhPYR1-3A* under saline stress (Fig. 6), pointing to the important roles of GhPYL9-5D and GhPYR1-3A in *Arabidopsis* seedling tolerance to salt and/or osmotic stress. Of note, the root growth of the overexpression lines of *GhPYR1-3A* in WT was suppressed by high concentrations of mannitol (Fig. 6). Similar results were found in transgenic plants expressing *OsPYL6* in rice [32], hinting that too strong ABA signals mediated by GhPYR1-3A and OsPYL6 likely negatively impact seedling tolerance to osmotic stress. As expected, ectopic expression of *GhPYL9-5D* and *GhPYR1-3A* clearly increased the root growth insensitivity of *112,458* to salt and osmotic stress, and salt tolerance of the mutant in soil (Fig. S7, S8), implying that GhPYL9-5D- and GhPYR1-3A-mediated ABA signals are essential for plant tolerance to salt and osmotic stress. Collectively, these data suggest that GhPYL9-5D and GhPYR1-3A might as ABA receptors fulfill important functions in responding to NaCl and osmotic stress during early development of *Arabidopsis* plants.

ABA is a key regulator of drought response in plants. Numerous ABA receptor genes have been demonstrated to increase plant drought tolerance after being overexpressed. These genes include *OsPYL3/5/6/7/9/10/11*, *AaPYL9*, tomato genes 6g050500 and 3g007310, *ZmPYL8/9/12*, *PtPYRL1/5*, *SlPYL9*, *TaPYL4*, *VyPYL9*, *MdPYL9*, *PePYL6/9*, *GhPYL9-11 A*, and *GhPYL10/12/26* [7, 14, 16, 24, 26, 27, 33, 38, 41, 45–49]. We found that the functions of cotton *GhPYL9-5D* and *GhPYR1-3A* were similar to those of the PYLs above (Fig. 8), supporting the notion that plant PYLs are conservative in positively modulating adaptation to dehydration stress. It has been observed that multiple PYLs described above are PYL9 homologs like OsPYL9, AaPYL9, ZmPYL9, SlPYL9, VyPYL9, MdPYL9, PePYL9, GhPYL9-11A and GhPYL9-5D [27–30, 33, 35, 42, 48], indicating that PYL9s may exert more important effects than other PYL members (for example PYR1s) in plant acclimation to drought stress.

In this report, knockdown of *GhPYL9-5D* and *GhPYR1-3A* by VIGS methods clearly enhanced the sensitivity of cotton seedlings to high concentrations of NaCl, mannitol and PEG (Fig. 10), suggesting that GhPYL9-5D and GhPYR1-3A positively regulate the response to salinity, osmotic stress and water deficit not only in *Arabidopsis* but also in cotton.

ABA controls stomatal closure in plants [5]. ABA-induced stomatal closure are clearly disrupted in *Arabidopsis* mutants *pyr1pyl1/2/4*, *112,458*, and *pyr1pyl1/2/3/4/5/7/8/9/10/11/12* [14, 16, 46], implying that most ABA receptors redundantly and positively regulate ABA-affected stomatal closure in *Arabidopsis*. In rice, knockout mutants of *OsPYL1/2/3/4/5/6/12* show strong insensitivity in ABA-triggered stomatal closure [24]. Consistently, transgenic plants overexpressing *Arabidopsis PYR1*/*RCAR11, PYL1*/*RCAR12, PYL2*/*RCAR14*, and *PYL3*/*RCAR13* are hypersensitive to ABA-stimulated stomatal closure [13]. The overexpression plants of *PtPYRL1* and *PtPYRL5* in *Arabidopsis* exhibit increased susceptibility to ABA-induced stomatal closure upon ABA treatment [45]. Constitutive overexpression of *AaPYL9-9* in *Artemisia* also significantly promotes ABA-elicited stomatal closure [35]. These results are in agreement with our findings about the roles of GhPYL9-5D and GhPYR1-3A in stomatal movement (Fig. 9), demonstrating that PYLs are crucial and conservative regulators of stomatal closure induced by ABA in plants.

Transcriptomic data revealed that *GhPYL9-5D*, *GhPYR1-3A* and their homologs in cotton were strongly expressed after treatment with high concentrations of PEG, NaCl and/or high and low temperature stress (Fig. S9). Moreover, GhPYL9-5D and GhPYR1-3A were co-expressed with many genes related to the signaling of redox, ABA and auxin (Fig. 11). These data imply that GhPYL9-5D and GhPYR1-3A regulate drought and salt tolerance of cotton possibly through affecting the expression of a number of stress- and hormone-related genes.

## Conclusions

GhPYL9-5D and GhPYR1-3A were localized in the cytoplasm and nucleus. They might as ABA receptors positively function in ABA-modulated seed germination, primary root growth and stomatal closure, as well as in response to water deficit, salt and osmotic stress likely through altering the expression of related genes in *Arabidopsis* and cotton. Yet, the underlying mechanisms are largely unknown. It deserved to study which GhPP2Cs, GhSnRK2s and transcriptional factors are regulated by GhPYL9-5D and GhPYR1-3A during ABA-affected growth and development and in response to various abiotic stresses in cotton. Nevertheless, these data will helpful for further investigating ABA signaling mechanisms in cotton in the future.

## Materials and methods

### Plant materials and growth conditions

Seeds of *Arabidopsis thaliana* WT plants (Col-0, CS70000, https://www.arabidopsis.org/servlets/Search?type=general&search_action=detail&method=1&show_obsolete=F&name=CS70000&sub_type=germplasm&SEARCH_EXACT=4&SEARCH_CONTAINS=1) were obtained from the Arabidopsis Biological Resource Center (ABRC) [50], and the seeds of *112,458* were kindly provided by Dr. Fuqiang Cui from Zhejiang A&F University in China [43]. The seeds of *112,458* and WT plants were sterilized, washed and sown on solid MS medium supplied with 3% (w/v) sucrose. After stratification at 4°C for 2 d, the seeds were germinated and the seedlings were grown in a growth chamber (day/night temperature of 21℃/18℃, light intensity of about 100 µmol m^− 2^ s^− 1^, 16 h light/8 h dark, and ∼70% relative humidity). Seeds of *G. hirsutum* TM-1 plants were kindly provided by Dr. Wuwei Ye from Institute of Cotton Research of Chinese Academy of Agricultural Sciences in China [51]. TM-1 seedlings were cultivated in a growth chamber with 28℃/26℃ day/night temperature, 200 µmol m^− 2^ s^− 1^ light intensity, 16 h/8 h light/dark cycle, and ∼50% relative humidity.

### Subcellular localization of GhPYL9-5D and GhPYR1-3 A

The full-length cDNA sequence information of *GhPYL9-5D* and *GhPYR1-3A* was obtained from COTTONMICS website (http://cotton.zju.edu.cn/index.htm). The coding DNA sequences of *GhPYL9-5D* and *GhPYR1-3A* were amplified by PCR from the TM-1 leaves using Phanta Max Super-Fidelity DNA Polymerase (Vazyme, cat no. P505-d1) (Initial denaturation, 95℃ for 3 min; denaturation, 95℃ for 15 s; annealing, 60℃ for 15 s; extension, 72℃ for 2 min; 35 cycles). They were cloned into the plant binary vector pCAMBIA1300-GFP to produce the complementation constructs *35S::GhPYL9-5D-GFP* and *35S::GhPYR1-3 A-GFP*. The primer sequences were listed in Table S1. About 20 µg plasmid DNA of *35S::GhPYL9-5D-GFP*, *35S::GhPYR1-3 A-GFP* or *35S::GFP* was used to transfect *Arabidopsis* leaf protoplasts following the method described previously [52]. Briefly, protoplasts were generated through the degradation of the leaves from 28-day-old WT seedlings using 1% (w/v) cellulose R10 and 0.4% (w/v) macerozyme. The protoplasts were then collected and incubated with the buffer solutions containing the construct DNA above using the PEG-Ca^2+^ transformation method. After incubation for 16 h, the GFP images and chloroplast autofluorescence in protoplasts were observed under a fluorescence light microscopy (Zeiss LSM510) at an excitation wavelength of 488 nm.

### Generation of *Arabidopsis* transgenic plants overexpressing *GhPYL9-5D* and *GhPYR1-3A*

To generate transgenic plants overexpressing *GhPYL9-5D* and *GhPYR1-3A*, the open reading frames of *GhPYL9-5D* and *GhPYR1-3A* were respectively amplified from the TM-1 leaves by PCR using the special primers in Table S1 (Initial denaturation, 95℃ for 3 min; denaturation, 95℃ for 15 s; annealing, 58℃ for 15 s; extension, 72℃ for 2 min; 35 cycles). Next, the amplified DNA fragments were cloned into the pCAMBIA1300 vector driven by CaMV 35S promoter. The *35S::GhPYL9-5D* and *35S::GhPYR1-3A* constructs were transformed into GV3101 strain cells of *Agrobacterium tumefaciens*, which were subsequently infiltrated into *Arabidopsis* WT and *112,458* mutant plants using the standard floral dip method. T1 transgenic seeds were selected based on hygromycin resistance, and T3 progeny homozygous plants were used in further studies.

### RNA extraction and gene expression analysis

Total RNA was extracted from seven-day-old *Arabidopsis* seedlings (100 mg) and two-week old TM-1 leaves (100 mg) using the RNA Plant Plus Reagen kit (Tiangen Biotech, cat no. DP441). RNA concentrations were determined with a Nanodrop-300 Spectrophotometer (Allsheng Instrument). Two micrograms of RNA in a 20 µL reaction mixture was applied for cDNA synthesis using the HiScript II cDNA Synthesis Kit (Vazyme, cat no. R211-01). RT-PCR experiments were performed to detect the mRNA abundances of *GhPYL9-5D* and *GhPYR1-3A* in the transgenic plants using the specific primers listed in Table S1. *Arabidopsis AtActin2* acted as the internal control. qRT-PCR experiments were carried out with the SYBR Color qPCR Master Mix (Vazyme, cat no. Q441-02) by a LightCycler® 480 II Real-Time PCR detection system (Roche). *Gossypium hirsutum GhUbiquitin7* and *Arabidopsis AtActin2* genes acted as the standard controls, respectively.

### Assay of seed germination and primary root elongation

At least fifty *Arabidopsis* seeds from WT and transgenic plants were sterilized, washed, and sowed on solid MS medium supplemented with 0, 0.3, 0.5 or 1 µM ABA, 0, 75 or 100 mM NaCl or 0, 200 or 250 mM mannitol. After stratification at 4℃ for 2 d, the seeds were germinated at a growth chamber described above. Germination is defined as the emergence of the radicle through the seed coats. We counted germinated seeds daily for 5 d. For greening ratio assay, seeds grown for 10 d after sowing before photographed, and expended green cotyledons were counted. For the measurement of primary root length, thirty seedlings of various lines (3-day-old) in solid MS medium were transferred to MS medium supplied with 0, 0.3, 0.5, 1, 2, 5 or 10 µM ABA, 0, 75 or 100 mM NaCl, or 0, 200 or 250 mM mannitol for 6–7 d. Then, the photographs were taken, and the elongation of primary roots was determined. The experiments repeated at least three times.

### Analysis of plant tolerance to drought and salt stress in soil

Drought tolerance of seedlings and leaf water loss were determined following the methods as described previously [53]. Briefly, the seedlings of 10-day-old *GhPYL9-5D* and *GhPYR1-3A Arabidopsis* overexpression lines grown in solid MS medium were transplanted into the nutrient soil (rich soil : vermiculite = 2 : 1, v/v) for another 10 d. Then, the plants were subjected to drought stress by withholding water for next 8 d, and rewatered for 2 d. The survival rates were examined after rewatering. The rosette leaves from 3-week-old plants grown in the nutrient soil were sampled, and placed on a scale to determine leaf water loss at the indicated time (0, 1, 2, 3, 4, 5 and 6 h) at 23℃.

For analyzing the roles of GhPYL9-5D and GhPYR1-3A in *Arabidopsis* tolerance to salt stress in soil, 2-week-old plants grown in the nutrient soil described above were watered with liquid MS medium containing 200 or 250 mM NaCl every other day for 14 d. The survival rates were calculated after treatment with 200 or 250 mM NaCl for 14 d.

### Stomatal aperture measurement

The aperture of stomata in leaf epidermal strips was monitored according to the method as described previously [54]. Briefly, epidermal strips were peeled from leaves of *Arabidopsis* plants, and put into 2-(N-morpholino) ethane sulfonic acid (MES)-KCl buffer solution (10 mM MES, 50 mM KCl, 100 µM CaCl_2_, pH 6.15). The strips were exposed to white light (150–200 µmol m^− 2^ s^− 1^) at 22 °C for about 3 h to stimulate stomata opening, and were then transferred to the MES-KCl buffer solution containing 0, 10 or 20 µM ABA for 30 min. The stomatal photographs were taken and stomatal aperture was measured under an inverted microscope (OLYMPUS IX73P1F). The experiments repeated at least three times, and more than 50 stomata for each replicate were assayed with the ImageJ software (https://imagej.nih.gov/ij/).

### Phenotype analysis of VIGS plants of *GhPYL9-5D* and *GhPYR1-3 A* in responding to PEG, NaCl and mannitol treatment

VIGS experiments were performed to knockdown genes *GhPYL9-5D* and *GhPYR1-3A* [55]. In brief, specific cDNA sequences of *GhPYL9-5D* (229 bp) and *GhPYR1-3A* (246 bp) were amplified using specific primers (Table S1), and cloned into the pTRV2 plasmid, respectively. The recombinant plasmids were then transformed into *A. tumefaciens strain* GV3101. Fully stretched cotyledons from seven-day-old cotton grown on vermiculite (watered with Hoagland’s nutrient solution) were inoculated with an equal amount of *Agrobacterium* mixed suspension containing one of pTRV2 recombinant vectors (*pTRV2::CLA1*, *pTRV2::GhPYL9-5D* and *pTRV2::GhPYR1-3A*) or empty vector (*pTRV2::00*) combined with pTRV1. After the injection for one week, the silenced cotton plants were identified by qPCR experiments applying the specific primers (Table S1), and transferred to Hoagland’s nutrient solution containing NaCl (200 and 300 mM), mannitol (200 and 300 mM) or PEG 6000 (15% and 20%) for 2 or 3 d. Photographs were then taken, and the fresh weight of seedlings was monitored after NaCl, mannitol and PEG treatments. All experiments repeated at least three times.

### Establishment of gene expression clustering and co-expression networks

The public RNA-seq data from previous research (PRJNA490626) [56] were used to analyze the expression profiles of *GhPYL*s in cotton under abiotic stresses, and in different tissues (http://cotton.zju.edu.cn/index.htm). Transcriptome analysis was carried out following the method described previously [57]. Differentially expressed genes were analyzed applying the R/edgeR package [58]. The 2 kb upstream sequences of *GhPYL9-5D* and *GhPYR1-3A* genes were committed to the PlantCARE (http://bioinformatics.psb.ugent.be/webtools/plantcare/html/) to retrieve the predicted imaginable cis-acting elements. The cis-acting elements were visualized, and gene expression heatmaps were drawn by the TBtools [59].

The R/WGCNA (Weighted gene co-expression network analysis) package was used to construct co-expression networks according to the method in previous studies [60]. The raw read count data were normalized using the logarithmic transformation method built in the DESeq2 software. After removing genes with zero expression or no differential expression among relevant samples, the remaining genes were clustered into network modules using the topological overlap measurement method. Genes were grouped by expression patterns with different clusters based on gene connectivity. Then, a similarity matrix was calculated by applying a power function (β) based on Pearson’s correlation between each pair of genes. The co-expression clusters were produced by dynamic Mods that keep the modules minimum size at least 30 genes. The hub genes were identified by the cytoHubba/Cytoscape software, and co-expression networks were displayed by the Cytoscape software [61, 62].

## Electronic supplementary material

Below is the link to the electronic supplementary material.

Supplementary Material 1

Supplementary Material 2

## Data Availability

The genome sequence and annotation files of *Gossypium hirsutum* L TM-1 were obtained from COTTONMICS website (http://cotton.zju.edu.cn/index.htm). RNA-Seq data in this study have been deposited at the National Center of Biotechnology Information (http://www.ncbi.nlm.nih.gov/) under the accessions PRJNA490626 (BioProject number: PRJNA490626, SRA number: SRP166405). The *Arabidopsis thaliana* WT plant seeds were from ABRC (CS70000), and TM-1 cotton seeds were provided by Dr. Wuwei Ye from Institute of Cotton Research of Chinese Academy of Agricultural Sciences in China. The *112,458* sextuple mutant seeds were provided by Dr. Fuqiang Cui from Zhejiang A&F University in China.

## Abbreviations

*112458*: *pyr1pyl1pyl2pyl4pyl5pyl8*
ABA: Abscisic acid
PEG: Polyethylene glycol 6000
PYL9-5D: Pyrabactin resistance like 9-5D
PYR1-3A: Pyrabactin resistance 1-3 A
ROS: Reactive oxygen species
WGCNA: Weighted gene co-expression network analysis
VIGS: Virus-induced gene silencing
