# Supplementary material for: GhPYL9-5D and GhPYR1-3 A positively regulate Arabidopsis and cotton responses to ABA, drought, high salinity and osmotic stress
Source: BMC Plant Biol. 2023 Jun 10;23:310. doi: 10.1186/s12870-023-04330-8 (PMC10257300; doi:10.1186/s12870-023-04330-8)
Supplement: Supplementary file 1 — Supplementary Material 1 [file 12870_2023_4330_MOESM1_ESM.doc]

**GhPYL9-5D and GhPYR1-3A positively regulate Arabidopsis and cotton responses to ABA, drought, high salinity and osmotic stress**

Yibin Wang^1^, Gaofeng Zhang^1^, Huimin Zhou^1^, Shanshan Yin, Yunxiang Li, Caixia Ma, Pengyun Chen, Lirong Sun, Fushun Hao*

**
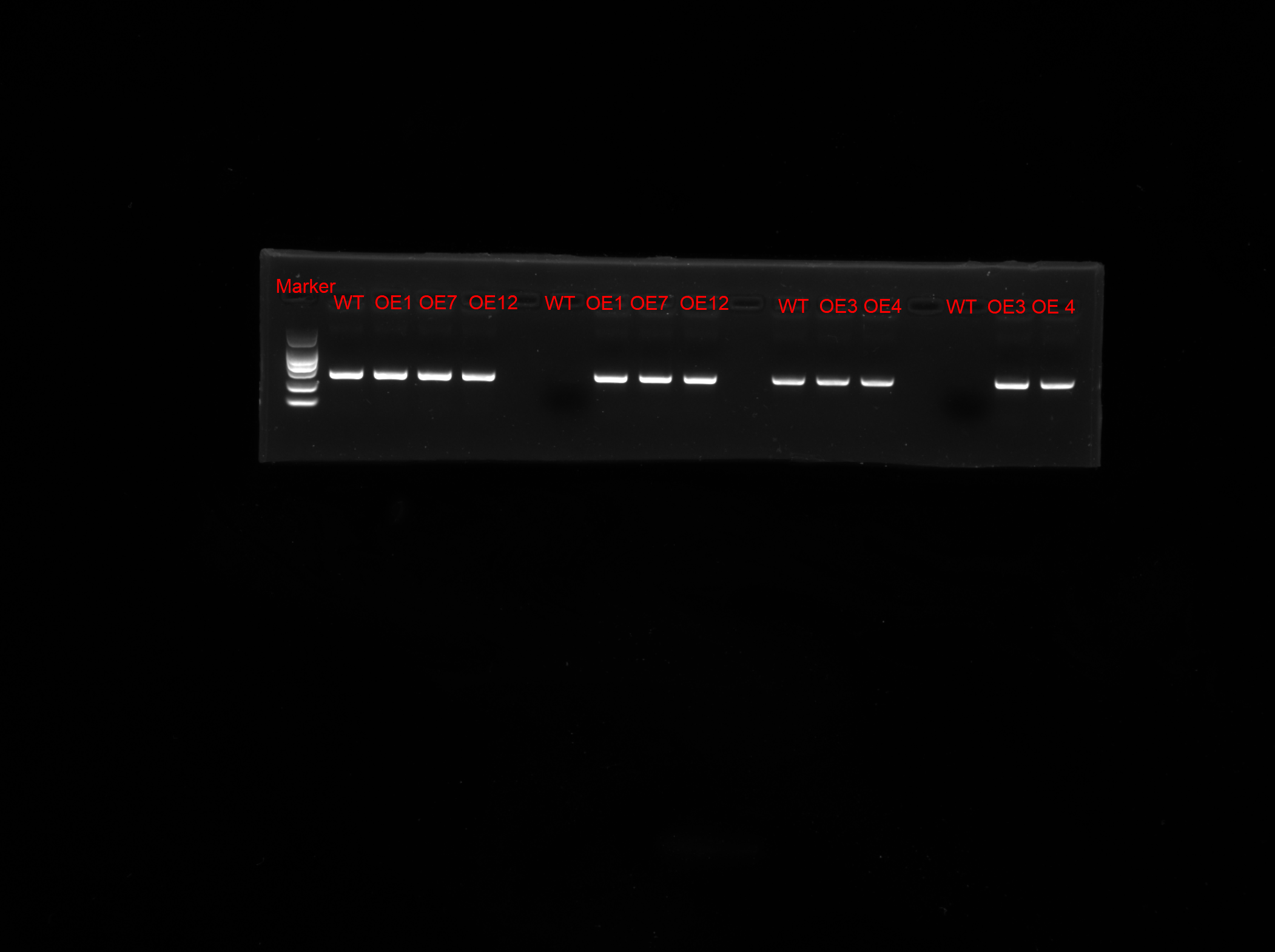

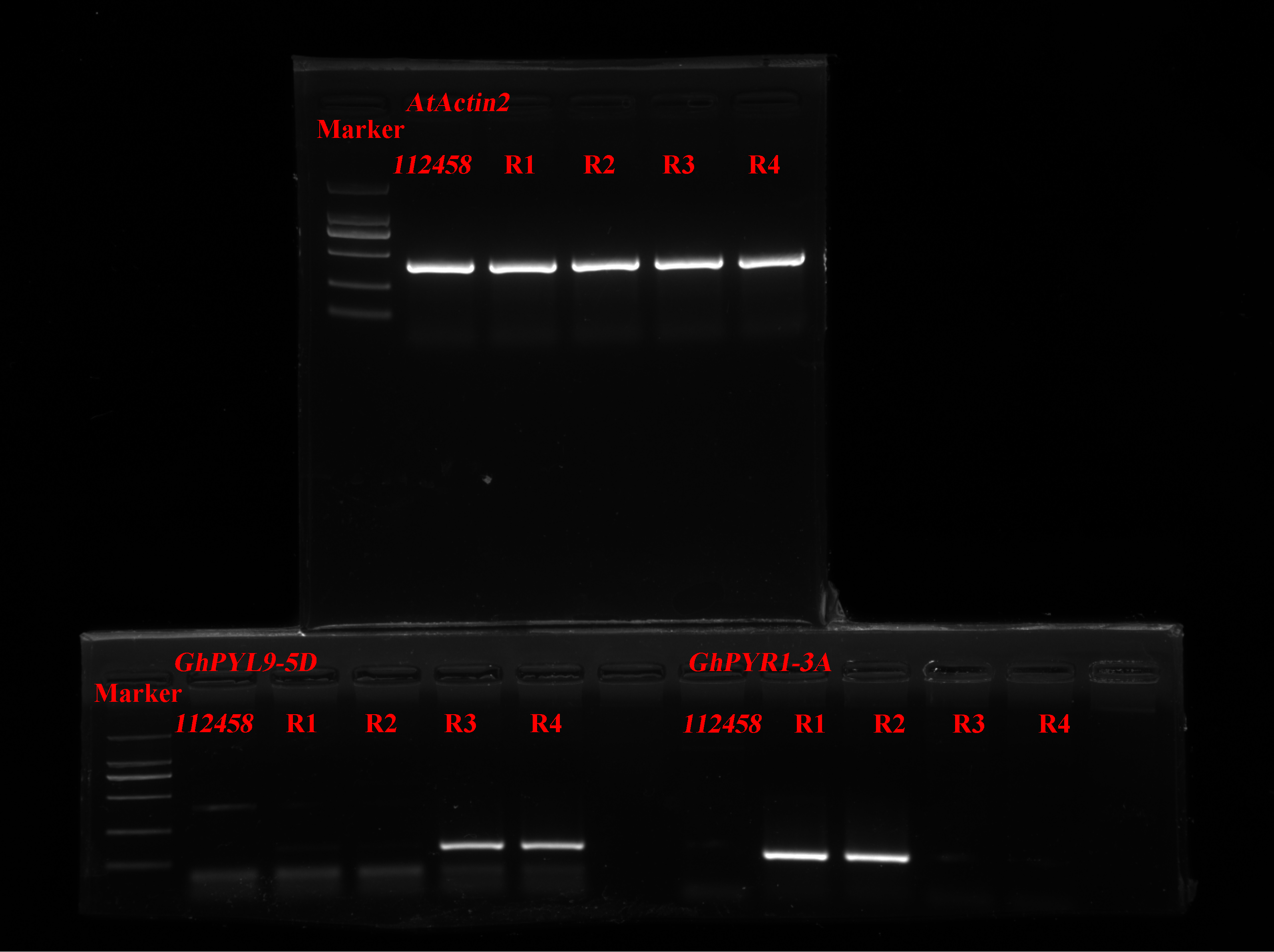
**

**Figure S1. RT-PCR analysis of transgenic Arabidopsis plants.**

The original images of agarose gels for *GhPYL9-5D* and *GhPYR1-3A*.


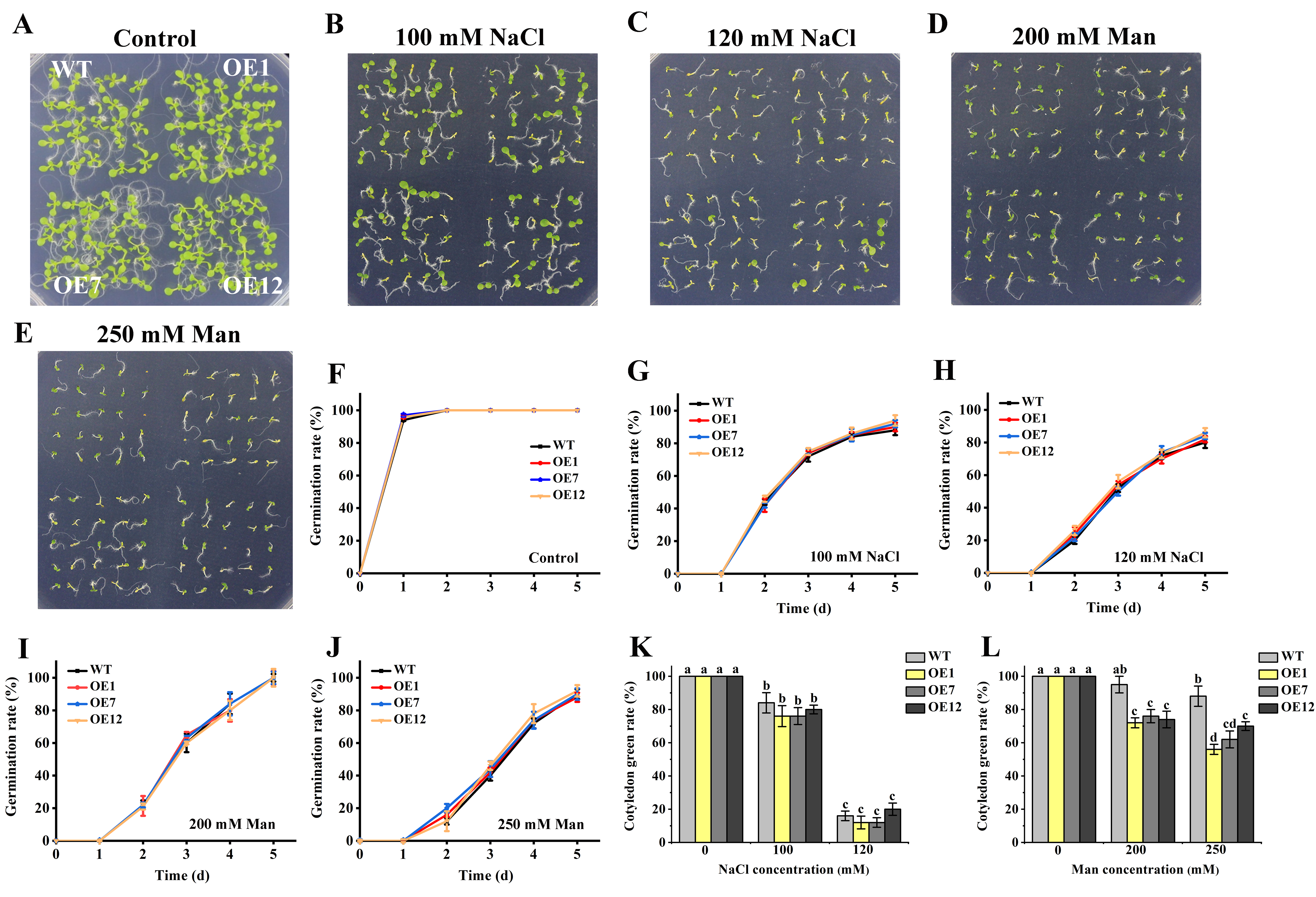


**Figure S2. Seed germination of WT, OE1, OE7 and OE12 in response to different concentrations of NaCl and mannitol.**

(A)-(E) Seeds of WT, OE1, OE7 and OE12 germinated in MS medium supplemented with 0 (Control), 100 mM NaCl, 120 mM NaCl, 200 mM mannitol (Man) and 250 mM mannitol for 7 d (at least 25 seeds for each genotype), respectively. (F)-(J) Seed germination rates of WT and the three OE lines in MS medium containing 0 (Control), 100 mM NaCl, 120 mM NaCl, 200 mM mannitol and 250 mM mannitol, respectively. (K) and (L) Cotyledon green rates of WT and the three OE lines above in MS medium with different concentrations of NaCl (0, 100 and 120 mM) and mannitol (0, 200 and 250 mM), respectively for 10 d. Data are mean ± SD (n ≥ 3). Different lowercase letters above the bars mean notable differences between two plants by one way ANOVA and Tukey’s HSD test (*P* < 0.05).


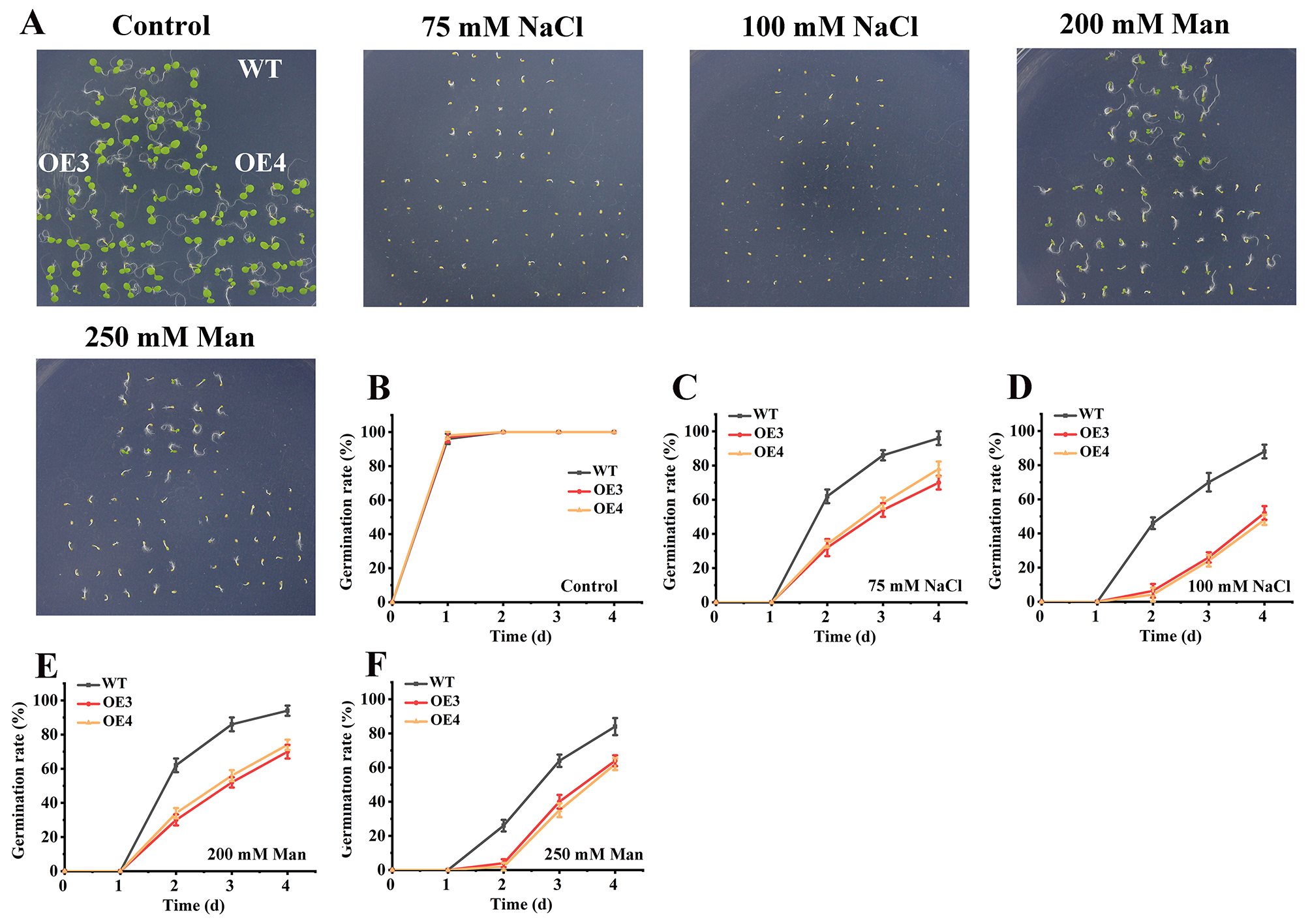


**Figure S3. Seed germination of WT, OE3 and OE4 under salt and osmotic stress.**

(A) Seeds of WT, OE3, and OE4 germinated in MS medium containing 0 (Control), 75 mM NaCl, 100 mM NaCl, 200 mM mannitol (Man) and 250 mM mannitol for 7 d (at least 25 seeds for each genotype), respectively. (B)-(F) Seed germination rates of WT and the two OE lines in MS medium with 0 (Control), 75 mM NaCl, 100 mM NaCl, 200 mM mannitol and 250 mM mannitol, respectively. Data are mean ± SD (n ≥ 3).


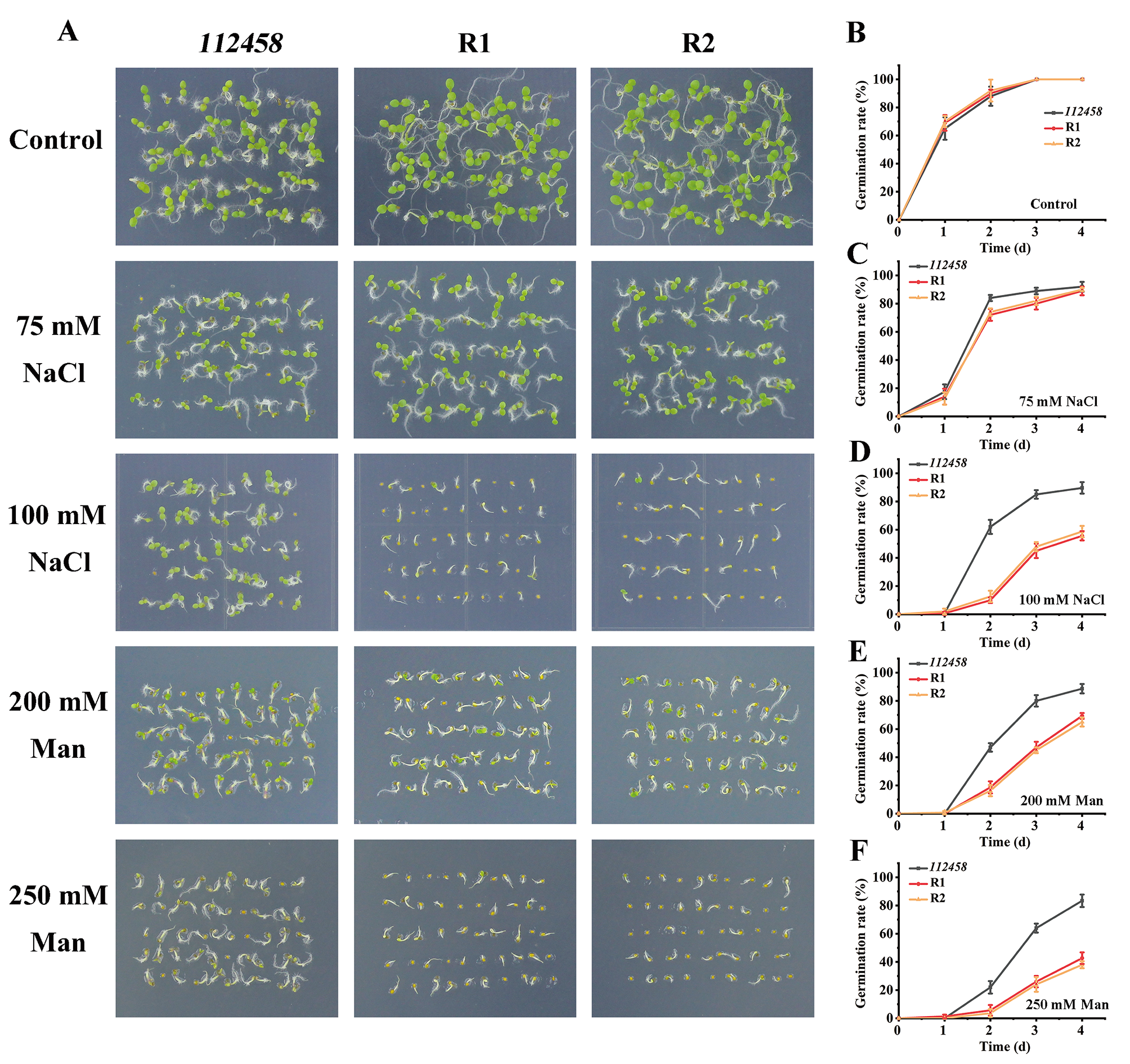


**Figure S4. Seed germination of *112458* mutant, R1 and R2 upon NaCl and mannitol stress.**

(A) Seed germination performances of *112458*, R1 and R2 in MS medium supplied with 0 (Control), 75 mM NaCl, 100 mM NaCl, 200 mM mannitol (Man) and 250 mM mannitol for 7 d (at least 50 seeds for each genotype), respectively. (B)-(F) Seed germination rates of the three plants above in MS medium containing 0 (Control), 75 mM NaCl, 100 mM NaCl, 200 mM mannitol and 250 mM mannitol, respectively. Data are mean ± SD (n ≥ 3)
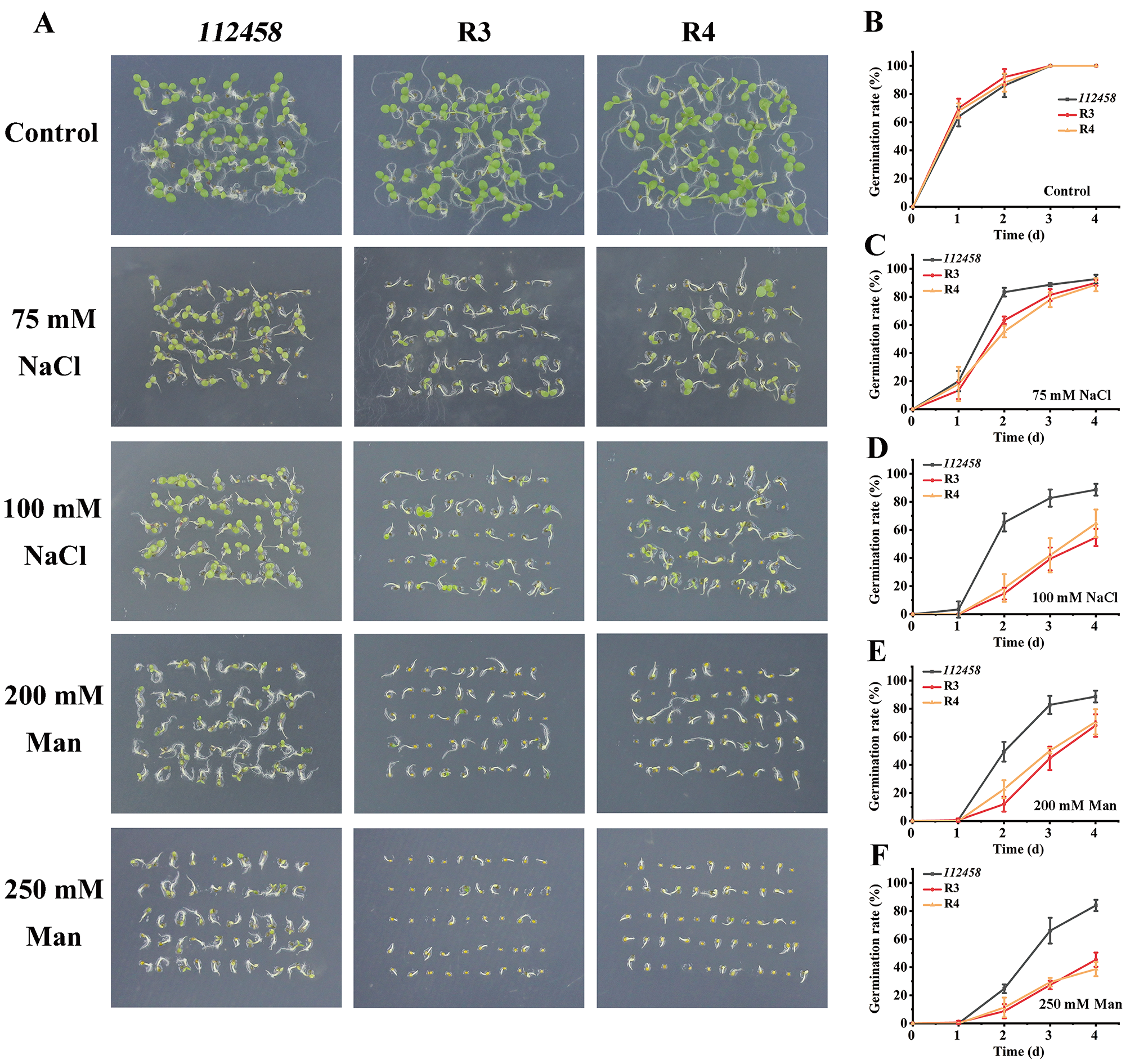


**Figure S5. Seed germination of *112458* mutant, R3 and R4 in responding to NaCl and mannitol stress.**

(A) Seed germination performances of *112458*, R3 and R4 in MS medium supplemented with 0 (Control), 75 mM NaCl, 100 mM NaCl, 200 mM mannitol (Man) and 250 mM mannitol for 7 d (at least 50 seeds for each genotype), respectively. (B)-(F) Seed germination rates of the three plants above in MS medium with 0 (Control), 75 mM NaCl, 100 mM NaCl, 200 mM mannitol and 250 mM mannitol, respectively. Data are mean ± SD (n ≥ 30).


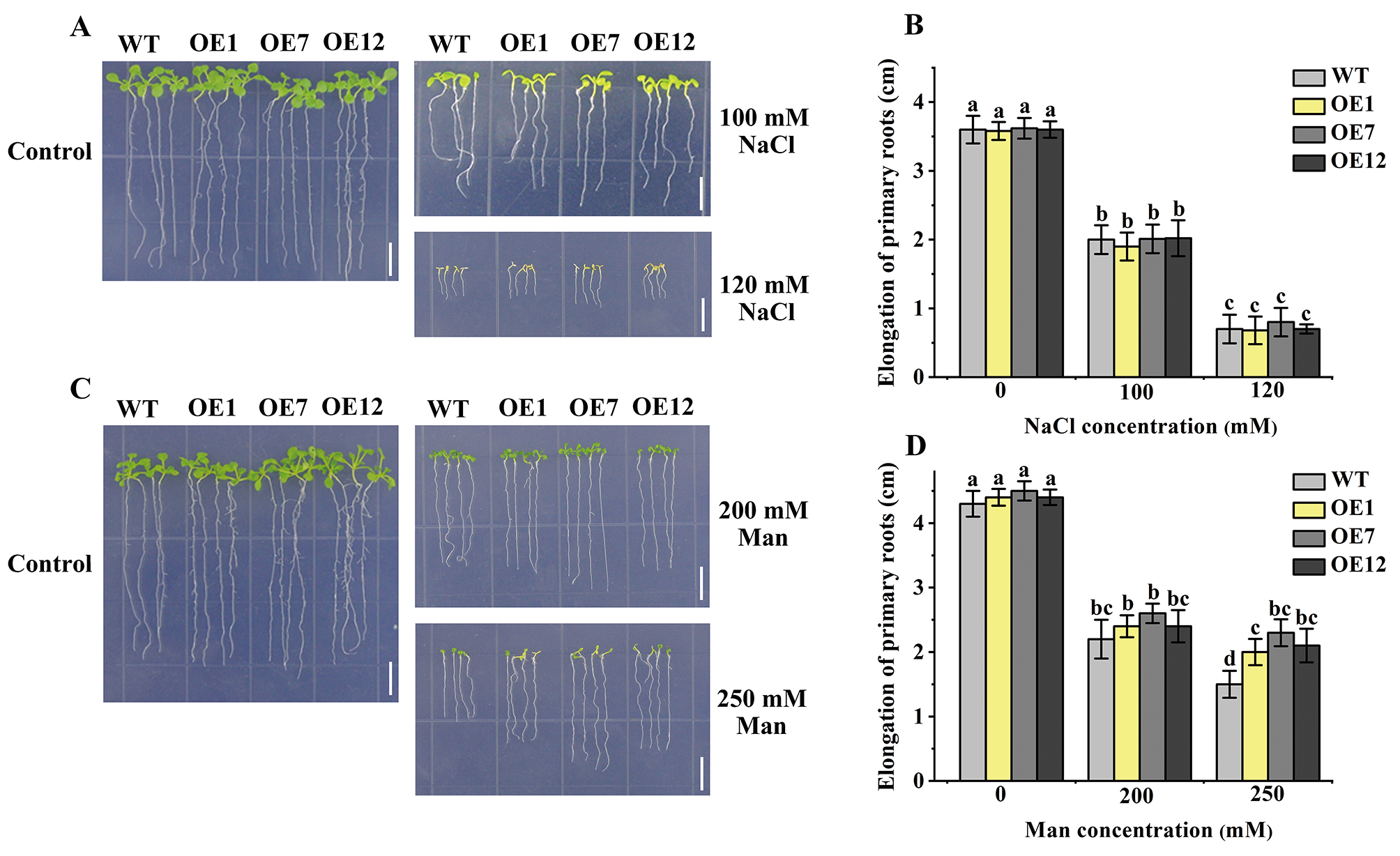


**Figure S6. Root growth of *GhPYL9-5D* overexpressors in response to NaCl and mannitol treatments.**

(A) and (C) Growth performances of OE1, OE7, OE12 and WT plants. Bar is 1 cm. (B) and (D) The increased primary root length of various lines. Three-day-old seedlings were transferred to MS medium without (Control) or with 100 mM NaCl, 120 mM NaCl, 200 mM mannitol (Man) or 250 mM mannitol for 7 d. Data are mean ± SD (n ≥ 30). Different lowercase letters above the error bars indicate significant differences between two plants by one way ANOVA and Tukey’s HSD test (*P* < 0.05).


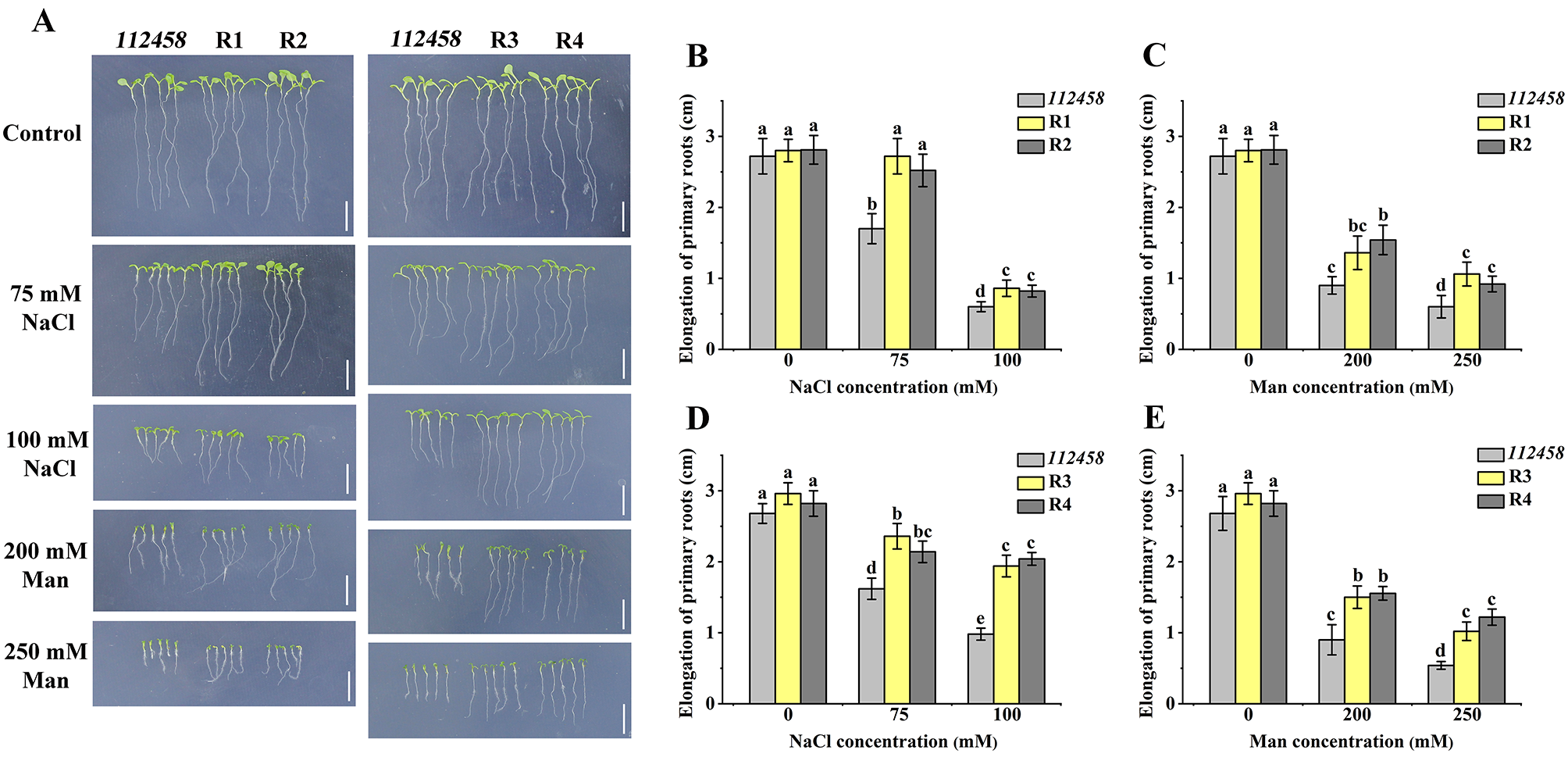


**Figure S7. Root growth of mutant *112458*, R1, R2, R3 and R4 under salt and osmotic stress.**

(A) Growth performances of all plants. Bar is 1 cm. (B) and (C) The increased primary root length of *112458*, R1 and R2 in response to different concentrations of NaCl and mannitol (Man), respectively. (D) and (E) The increased primary root length of *112458*, R3 and R4 after challenged by different concentrations of NaCl and mannitol, respectively. Three-day-old seedlings were transferred to MS medium supplied without (Control) or with 75 mM NaCl, 100 mM NaCl, 200 mM mannitol or 250 mM mannitol for 5 d. Data are mean ± SD (n ≥ 30). Different lowercase letters above the error bars reveal significant differences between two lines by one way ANOVA and Tukey’s HSD test (*P* < 0.05).


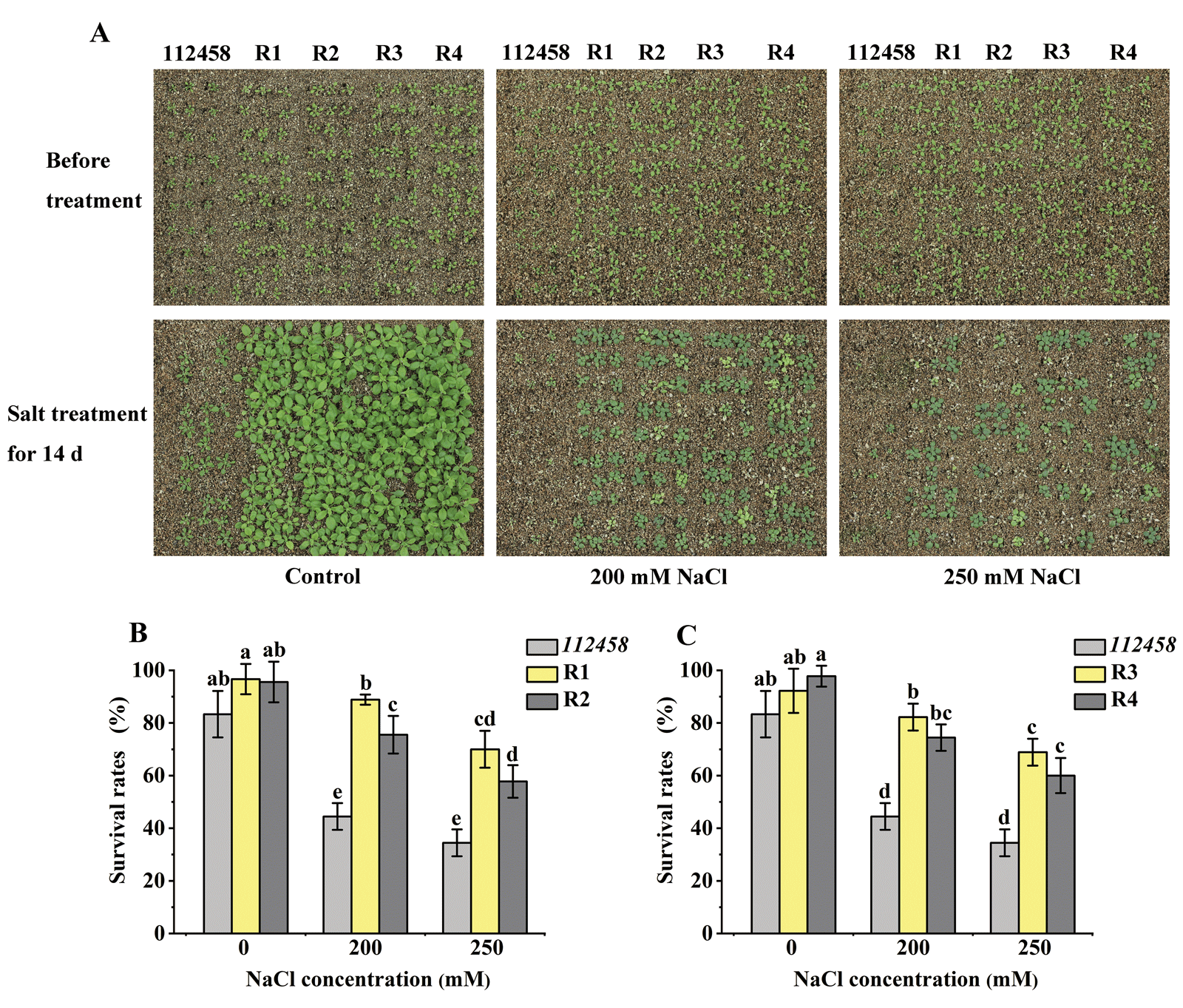


**Figure S8. GhPYL9-5D and GhPYR1-3A conferred salt tolerance to *112458* mutant.**

(A) Growth performances of *112458* mutant, R1, R2, R3 and R4. (B) and (C) Survival rates of overexpressors of *GhPYL9-5D* and *GhPYR1-3A* in *112458*, respectively, under salt stress. Four-week-old plants were treated with different concentrations of NaCl (0, 200 and 250 mM) for 14 d. Data are mean ± SD (n ≥ 3). Different lowercase letters above the error bars represent that the values between two lines were significantly different by one way ANOVA and Tukey’s HSD test (*P* < 0.05).


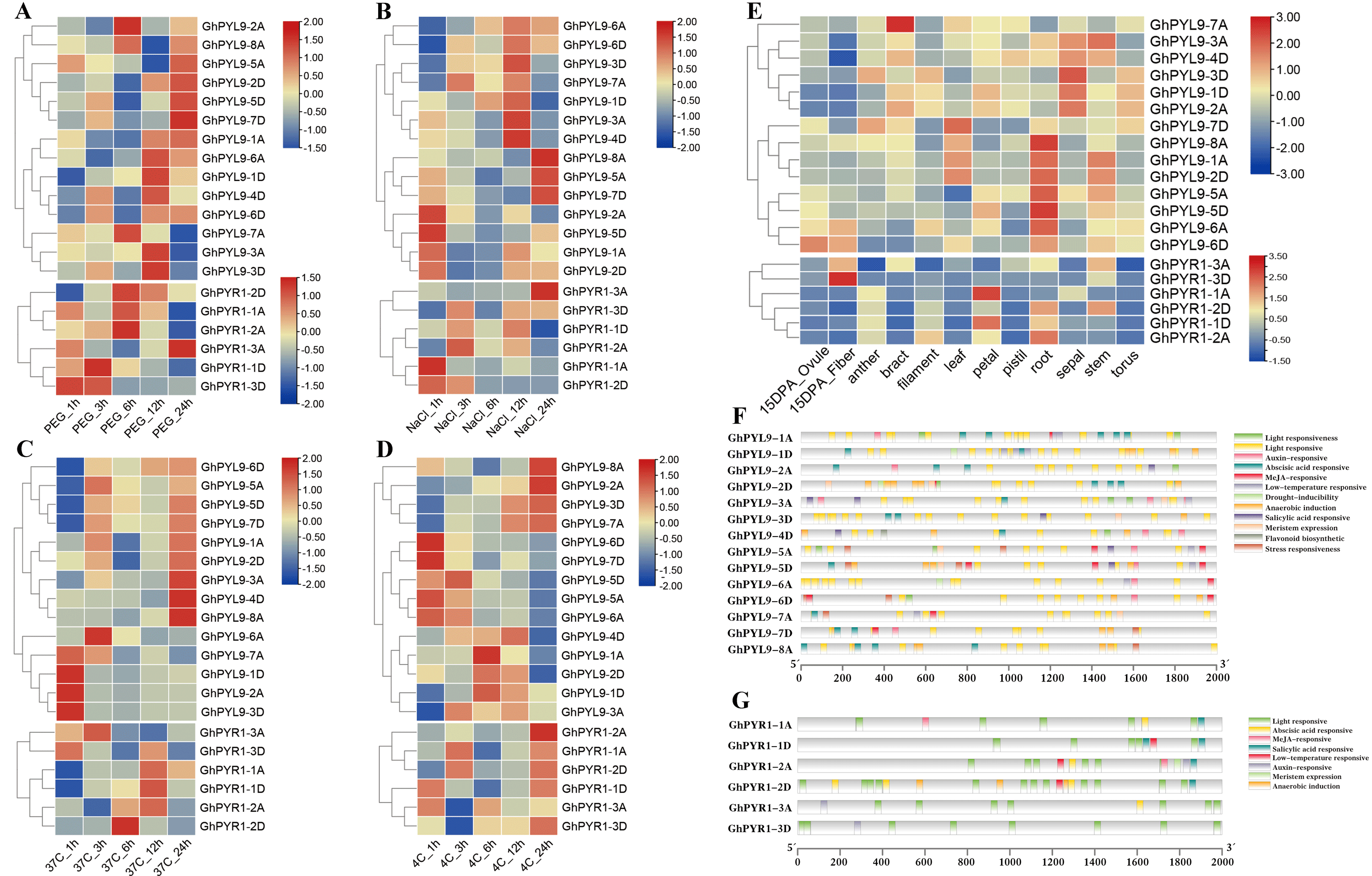


**Figure S9. Expression patterns of** ***GhPYL9-5D*, *GhPYR1-3A*, and their orthologs upon stresses and in tissues as well as the cis-elements of these genes.**

(A)-(D) Expression profiles of 14 *GhPYL9s* and *6 GhPYR1s* in responding to 20% PEG treatment, salt stress (400 mM NaCl), high temperature (37℃) and low temperature (4℃), respectively. (E) Expression of 14 *GhPYL9s* and 6 *GhPYR1s* in different tissues of cotton. (F) and (G) The predicted cis-elements in gene promoters of 14 *GhPYL9s* and 6 *GhPYR1s*, respectively.
