# Supplementary material for: GhPYL9-5D and GhPYR1-3 A positively regulate Arabidopsis and cotton responses to ABA, drought, high salinity and osmotic stress
Source: BMC Plant Biol. 2023 Jun 10;23:310. doi: 10.1186/s12870-023-04330-8 (PMC10257300; doi:10.1186/s12870-023-04330-8)
Supplement: Supplementary file 2 — Supplementary Material 2 [file 12870_2023_4330_MOESM2_ESM.docx]

**Table S1: Primers used in this study.**

| Primer Name | Sequence (5'-3') |
| --- | --- |
| PYL9-5D-GFP--F | cggggtaccATGGTGACCAATAATTATA |
| PYL9-5D-GFP-R | gggatccCATTCTTTCGATCGGCTC |
| PYR1-3A-GFP-F | ggggtaccATGGCAGTCTCAAAACC |
| PYR1-3A-GFP-R | cgggatccTGAATTATTACCGTCATT |
| pTRV2- GhPYL9-5D-F | agaaggcctccatggggatccAGCATGAGGATTGTTGGAGG |
| pTRV2- GhPYL9-5D-R | tgtcttcgggacatgcccgggGATCCTGAACAGCCAAATGC |
| pTRV2- GhPYR1-3A-F | agaaggcctccatggggatccGCACCGAGAGACTGGATATTTT |
| pTRV2- GhPYR1-3A-R | tgtcttcgggacatgcccgggCAGACGCTAGCTTTTGCAAGTT |
| PYL9-5D-OE-F | cggggtaccATGGTGACCAATAATTATA |
| PYL9-5D-OE-R | gggatccCATTCTTTCGATCGGCTC |
| PYR1-3A-OE-F | ggggtaccATGGCAGTCTCAAAACC |
| PYR1-3A-OE-R | cgggatccTGAATTATTACCGTCATT |
| AtActin2-F | AATTACCCGATGGGCA |
| AtActin2-R | TCATACTCGGCCTTGGA |
| PYL9-5D-q-F | TCGCTCGTTAAACACATCAAAG |
| PYL9-5D-q-R | GAACAGCCAAATGCTCTGAAAC |
| PYL1-3A-q-F | AAACCACCACTCACCACCTCAC |
| PYL1-3A-q-R | TCTTCCTCCGTATTACCTTCCG |
| GhUBQ7-F | GAAGGCATTCCACCTGACCAAC |
| GhUBQ7-R | CTTGACCTTCTTCTTCTTGTGCTTG |
